# Genomic Structural Equation Modeling Provides an Initial View of the Genetic Architecture Related to Type 1 Gaucher Disease

**DOI:** 10.1155/humu/1692822

**Published:** 2026-04-15

**Authors:** Shijie Ren, Mingmin Du, Jingyuan Liu, Boyu Li, Jianping Liu, Xiaomeng Lang

**Affiliations:** ^1^ Graduate School, Hebei University of Chinese Medicine, Shijiazhuang, Hebei Province, China, hbtcm.edu.cn; ^2^ Department of Gastroenterology, The First Affiliated Hospital of Hebei University of Chinese Medicine, Shijiazhuang, Hebei Province, China

**Keywords:** genetic structure, genome-wide association study, genomic SEM, missense mutation, Type 1 Gaucher disease

## Abstract

The genetic architecture underlying traits associated with Type 1 Gaucher disease (GD1) remains insufficiently explored. We integrated genomic structural equation modeling and multiple post–genome‐wide association study (GWAS) methodologies to prioritize candidate SNPs associated with GD1‐related variation, identifying 15 loci with strong statistical support. Subsequently, diverse transcriptome‐wide association approaches were employed to pinpoint susceptibility gene signals strongly correlated with GD1. For selected candidate genes, we explored the potential structural consequences of missense variants using integrated structure prediction, molecular dynamics simulations, and AI‐based thermodynamic stability analyses. These analyses suggested that the mutations may alter protein structure and dynamics, with possible consequences for protein stability and biological function. Next, we screened a large set of publicly available traits to identify GD1‐related factors and biomarkers with potential relevance. Finally, a summary data–based polygenic risk score (PRS) was utilized to examine risk associations between 22 autosomes and GD1. Collectively, by modeling a GD1‐related phenotype without direct prior measurement, this study provides an initial overview of the shared genetic architecture associated with GD1.

## 1. Introduction

Type 1 Gaucher disease (GD1) is a lysosomal storage disorder caused by mutations in the GBA1 gene, characterized by irreversible glucosylceramide accumulation in macrophages that triggers systemic pathology, including hepatosplenomegaly, anemia, osteolytic bone destruction, and dysregulated lipid metabolism [1]. With the global expansion of precision diagnostics, GD1 detection rates continue to rise—particularly among Ashkenazi Jewish populations with a high carrier frequency—establishing GD1 as a critical challenge in inherited metabolic diseases [2]. Although enzyme replacement therapy ameliorates select symptoms, the genetic regulatory networks and mechanisms underlying phenotypic heterogeneity remain poorly defined [3]. Current evidence implicates impaired lysosomal autophagy and neuroinflammatory inflammasome activation as core drivers of disease progression, yet these pathways incompletely explain interindividual phenotypic variability [4, 5].

The pathogenic mechanisms of GD1 exhibit considerable complexity. Enabled by advancements from the Human Genome Project, genome‐wide association studies (GWASs) have established the significant heritability of traits such as hepatosplenomegaly [6]. Key susceptibility loci for these traits have been identified through GWAS, including single‐nucleotide polymorphism (SNP) rs76763715 near the GBA gene, which shows a robust association with hepatosplenomegaly [7]. Specific SNPs in the HFE gene (e.g., rs1799945) correlate with anemia development, particularly in GD1 patients with iron overload [8]. Proinflammatory cytokine–associated SNPs (e.g., rs1800629 in TNF‐*α* and rs1800796 in IL‐6) may contribute to osteoporosis and osteolytic destruction [9, 10]. Notably, copy number variations in UGT2B17 are significantly linked to reduced bone mineral density and elevated fracture risk [11]. GWAS‐identified variants in the APOE gene (rs429358 and rs7412) modulate dysregulated lipid metabolism, especially hypercholesterolemia and hypertriglyceridemia [12]. These findings underscore the independent genetic regulation of distinct GD1 phenotypes. However, the precise mechanisms driving the co‐occurrence of hepatosplenomegaly, anemia, osteolytic destruction, and metabolic dysregulation in individual patients remain elusive [13]. Current GWAS discoveries remain understudied within the GD1 pathophysiological context.

To unravel this complexity, we integrated diverse genetic analytical tools, robust correlation exploration methodologies, high‐precision structural predictions, and protein model–based dynamic simulations. This framework was designed to generate hypotheses regarding the shared genetic basis of GD1‐related traits and to improve our understanding of their relationships with multiple comorbid conditions. Specifically, this study focused on GD1‐associated loci and chromosomal regions to highlight candidate genes and pathways that may merit further investigation. These analyses may contribute to a broader understanding of GD1‐related biology and provide a basis for future mechanistic and translational studies.

**Figure 1:**
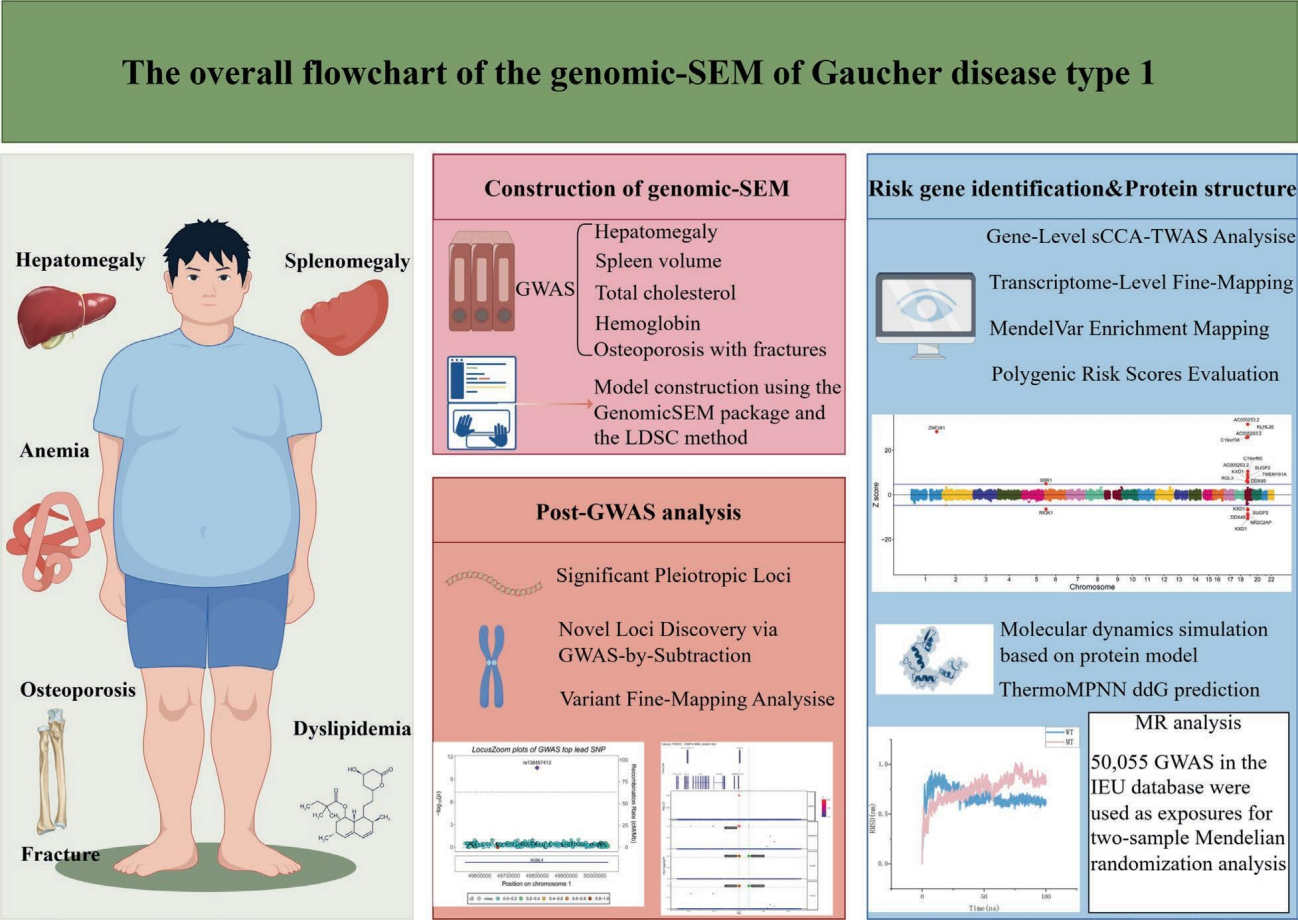
Construction and analysis process of genomic SEM for GD1.

In this study, given the lack of direct large‐scale genetic measurements for GD1, we applied genomic structural equation modeling (SEM) to published GWAS summary statistics of GD1‐related traits and biomarkers. This framework integrates genome‐wide association data with SEM to characterize shared genetic architecture among correlated traits while accounting for potential sample overlap and pleiotropic effects. By analyzing GD1‐related summary statistics, we estimated SNP associations with a GD1‐related latent construct rather than with directly measured clinical GD1 itself. We then used integrative downstream analyses to prioritize candidate loci, genes, and pathways that may contribute to this shared genetic signal. Although GD1 is influenced by genetic, environmental, and stochastic factors and our approach cannot fully resolve all interactions within GD1‐related biology, this framework provides an exploratory strategy for investigating a phenotype that is not directly measured. Finally, we conducted large‐scale association analyses to build a broad map of traits related to the GD1‐associated latent phenotype. This resource may serve as a reference for future mechanistic studies and clinical hypothesis generation. The overall workflow is depicted in Figure 1.

## 2. Materials and Methods

### 2.1. Sources of GWAS Data

Five GWAS summary–level datasets were utilized for genomic SEM analysis. These datasets represent independent GWASs closely associated with GD1. The summary statistics were sourced from FinnGen R12, IEU OpenGWAS, UK Biobank (UKB), and previously published GWAS investigations. The included GD1‐related phenotypes encompassed hepatomegaly, spleen volume, total cholesterol, hemoglobin levels, and osteoporosis with pathological fractures. There are reasons for choosing these five phenotypes. Hepatomegaly and spleen volume directly reflect the degree of accumulation of lipid‐filled macrophages in the visceral organs. Total cholesterol indicates systemic lipid metabolism disorders. Hemoglobin levels can be used as evaluation indicators for bone marrow infiltration and hemocytopenia. Osteoporosis reflects the characteristic bone destruction of GD1. All GWASs received approval from their respective ethics committees and obtained participants′ informed consent. Rigorous quality control procedures were applied to all datasets. Detailed characteristics of these GWASs are provided in Table S1.

### 2.2. Quality Control of GWAS

We initially excluded low‐quality samples with missing rates exceeding 5%, followed by removal of the major histocompatibility complex (MHC) region (chr6: 25,000,000–35,000,000 base pairs) due to its genetic diversity and structural complexity, thereby ensuring analytical accuracy and robustness. For GD1 summary statistics preparation, default quality control standards were uniformly applied to filter SNPs across all five input GWAS datasets; using the 1000 Genomes Phase 3 European reference panel, we excluded SNPs exhibiting minor allele frequency (MAF) < 0.01, effect size estimates of zero, strand mismatches, or allele inconsistencies with the reference panel. The incorporated GWASs were obtained from different databases and research consortia. We attempted to minimize potential sample overlap by selecting datasets from distinct sources. However, because the analyses were based on publicly available summary statistics, strict nonoverlap at the individual‐sample level could not be fully confirmed.

### 2.3. Construction of Genomic SEM

The genomic SEM framework was implemented in R using the GenomicSEM package (v0.0.5) [14], through which SEM‐based GWAS analysis of five input summary datasets enabled investigation into the latent genetic susceptibility of GD1; notably, genomic SEM represents a novel analytical model that explores the underlying architecture of target traits by integrating multiple variables, with inherent robustness to sample overlap and sample size imbalances thus enhancing model accuracy, while specifically distinguishing local variants affecting individual phenotypes from broad‐susceptibility factors.

The complete genomic SEM workflow comprised two sequential phases. In the first phase, leveraging generated GWAS summary statistics for GD1 and applying a multivariate extension of linkage disequilibrium (LD) score regression (LDSC), we estimated the empirical genetic covariance matrix and corresponding sampling covariance matrix. This generated an empirical genetic covariance matrix across five GD1‐related traits, which served as input for the SEM common factor model. The number of filtered SNPs and heritability estimates for each trait are detailed in Table S2.

In the second phase, an SEM model was specified to minimize discrepancies between the hypothesized covariance structure and the empirically estimated covariance matrix derived from Phase 1. This procedure enabled the identification of latent genetic factors underlying the five GD1‐associated traits. A single‐factor model was consequently fitted and evaluated using four goodness‐of‐fit indices: comparative fit index (CFI), model chi‐square (*χ* ^2^), Akaike information criterion (AIC), and standardized root mean square residual (SRMR) (Tables S3 and S4). By incorporating appropriate common factor SEM specifications, individual autosomal SNP associations were integrated with genetic and sampling covariance matrices, permitting genome‐wide analysis of shared covariance across the five GD1 trait datasets. Finally, to verify appropriate modeling of SNP associations within the genomic SEM framework, SNP heterogeneity statistics were computed. SNPs with Cochran′s *Q* statistic *p* values < 0.05 were excluded from subsequent analyses.

### 2.4. Multilevel Evaluation of Genomic SEM

To verify genomic SEM′s robustness beyond conventional goodness‐of‐fit tests, we employed LDSC for stability assessment through comprehensive analysis of model fit metrics—including mean *χ* ^2^, genomic control lambda (*λ* GC), maximum *χ* ^2^, heritability (*h* ^2^), intercept, and outlier ratio (ratio = (intercept − 1)/(mean *χ* ^2^ − 1)); in addition, quality control parameters where SNPs with missing values, INFO scores < 0.9, or MAF < 0.01 were retained, while those exhibiting invalid *p* values or strand‐ambiguous alleles were systematically excluded.

### 2.5. Define Genomic Loci and Identify Novel Variants

Functional Mapping and Annotation (FUMA) was utilized for analyzing genomic loci, annotating lead SNPs associated with GD1 that exhibited low LD (*r* ^2^ < 0.1) and genome‐wide significance (*p* < 5 × 10^−8^) [15]. Subsequently, these GD1‐associated lead SNPs and loci were compared with previously reported SNPs/loci from univariate GWAS to explore pleiotropic effects, cross‐referencing established associations (*p* < 5 × 10^−8^) in the GWAS Catalog. FUMA was then applied for risk gene locus analysis within the GD1 GWAS framework at a significance threshold of *p* < 5 × 10^−8^, followed by Multi‐marker Analysis of GenoMic Annotation (MAGMA) for post‐GWAS assessment of variant–disease associations with false discovery rate correction (FDR corrected *p* < 0.05). Finally, the GWAS‐by‐subtraction approach was implemented, comparing genomic SEM–identified loci against single‐input GWAS loci (*p* < 5 × 10^−8^) to exclude known findings, thereby enhancing discovery of novel high‐utility loci and refining genetic precision and power.

### 2.6. Fine Mapping

To identify causal variants for GD1, we implemented fine‐mapping analyses using SuSIE (Sum of Single Effects) and FINEMAP through the echolocatoR v.2.0.3 package—both tools designed to pinpoint the most probable causal variants underlying target phenotypes—where 250 kb windows centered on lead SNPs were delineated to compute posterior inclusion probabilities (PIPs) for all regional SNPs, defining credible sets at a 0.95 probability threshold and generating consensus credible sets via “consensusNP” (codetected variants by both methods) with mean PIP (PIP = 1 when both SuSIE and FINEMAP PIP > 0.95). The reference panel is the Phase 3 dataset of the 1000 Genomes Project, and the population is European.

### 2.7. Transcriptome‐Wide Association Study (TWAS)

Following causal variant identification, we conducted TWASs to prioritize genes associated with GD1 by analyzing gene expression–phenotype relationships, implementing a cross‐tissue FUSION methodology—a sparse canonical correlation analysis (sCCA)–based approach—that leveraged precomputed expression quantitative trait locus (eQTL) weights for 37,920 genomic regions from GTEx v8 data to model tissue–specific expression associations [16]; genes with *p* < 0.05 were subsequently advanced for fine mapping of gene sets (FOCUS), a TWAS‐specific causal inference tool that calculates PIP for gene–trait causality (thresholded at PIP > 0.8), enabling prioritization of genes exhibiting significance in both FUSION and FOCUS analyses as high‐confidence causal candidates. In addition, MendelVar was used to perform gene enrichment analysis.

### 2.8. Protein Structural Alterations and Molecular Dynamics Simulations

Missense mutations, defined as single‐nucleotide substitutions causing amino acid alterations, typically disrupt protein structure and function. We therefore performed AlphaFold3‐based structural prediction and molecular dynamics simulations for selected TWAS‐prioritized genes to explore the possible structural consequences of missense variants. Specifically, missense mutations rs2102483805 (ZNF281) and rs200129596 (RIOK1) were analyzed. The substitution site was identified through Ensembl, and the Ensembl HGVS selected for rs2102483805 was ENSP00000356322.1:p.Val280Gly, while rs200129596 was selected Ensembl HGVS ENSP00000545595.1:p.Phe384Val. Wild‐type (WT) protein sequences (UniProt IDs: Q9Y2X9 for ZNF281 and Q9BRS2 for RIOK1) were retrieved from UniProt [17]—publicly accessible databases providing free sequence downloads. AlphaFold3, a state‐of‐the‐art deep learning algorithm, predicts tertiary protein structures with unprecedented speed and accuracy compared to traditional methods (e.g., X‐ray crystallography), particularly excelling in modeling mutational impacts [18]. Mutant (MT) protein sequences were submitted to AlphaFold3′s online platform (https://alphafoldserver.com) for structural prediction and subsequent analysis.

Next, we conducted in vivo protein simulations for both WT and MT forms of ZNF281 and RIOK1 using GROMACS 2024.5 to analyze their dynamic behavior and stability in solution. Molecular simulations were accelerated by an NVIDIA GeForce RTX 4090 GPU on an Ubuntu 24.04 LTS operating system, leveraging Linux′s stable computational environment optimized for efficient parallel processing of large‐scale tasks. Key simulation parameters included the AMBER14SB force field—widely adopted for accurately modeling amino acid residue interactions—for protein parameterization [19], paired with the TIP3P water model to simulate aqueous environments around biomolecules [20]. Protein topology files were generated using pdb2gmx, followed by system setup with editconf to position proteins within a cubic box (1.0 nm minimum boundary clearance). Solvation was performed via the solvate command to ensure uniform water distribution, while Genion added Na^+^/Cl^−^ ions to maintain charge neutrality at physiological concentration (0.15 M NaCl). Energy minimization (1000 steps) employed the Steepest Descent algorithm (max step size: 0.01 nm) to eliminate structural clashes. The system then underwent equilibration: 100 ps under constant temperature and volume conditions, followed by 100 ps under constant temperature and pressure conditions. Production molecular dynamics simulations ran for 100 ns (constant temperature and pressure conditions, 300 K and 1.0 atm) with a 2‐fs time step and SHAKE constraints on hydrogen bonds. Resulting trajectories were reserved for analysis and visualization.

### 2.9. Free Energy Change (ddG) Prediction Using the AI Model ThermoMPNN

To further assess the potential effects of these mutations on protein stability, ThermoMPNN was used to estimate mutation‐associated changes in thermal stability. Leveraging deep learning trained on extensive datasets of protein structures, mutation types, and corresponding thermal stability profiles, ThermoMPNN effectively predicts residue‐specific mutation effects on protein thermostability [21]. The model was executed within a Google Colab environment for efficient training and prediction. After configuring the Colab workspace for ThermoMPNN, the PDB files of WT structures were uploaded. Using default PyTorch framework parameters, ThermoMPNN modeled amino acid substitutions to compute postmutation stability variations and melting temperature (Tm) shifts across different temperatures.

### 2.10. Biomarker and Risk Factor Annotation Analysis

To identify associations between unmeasured GD1 GWAS data and pre‐existing diseases/biomarkers, we conducted large‐scale association analyses. Using the MendelianRandomization R package (v.0.10.0), we performed Mendelian randomization (MR) analyses with 50,055 phenotypes from the IEU OpenGWAS as potential exposures. Results underwent FDR correction, with FDR corrected *p* < 0.05 set as the significance threshold.

### 2.11. Polygenic Risk Scores (PRSs) Based on Summary Data

PRSs were calculated using summary statistics from GWASs to evaluate the genetic contributions of distinct chromosomal regions to GD1 pathogenesis. This analysis employed polygenic risk score with continuous shrinkage (PRS‐CS), a Bayesian regression–based tool that estimates posterior SNP effect sizes by integrating GWAS summary data with external LD reference panels, thereby generating polygenic risk metrics.

## 3. Results

### 3.1. Structural Equation Model Fit Assessment

**Figure 2:**
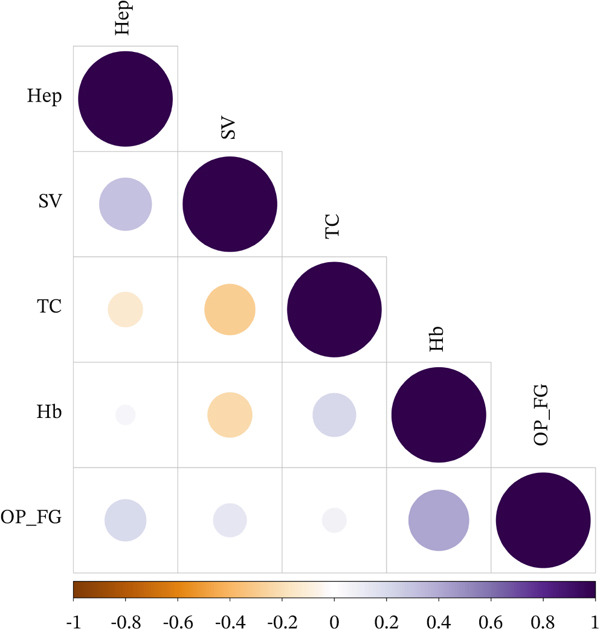
Genetic correlation matrix for GD1. The size and color depth of the circles represent the strength of the correlation between traits. Yellow and purple represent values ranging from −1 to 1, respectively. Hep represents hepatomegaly, SV represents spleen volume, TC represents total cholesterol, Hb represents hemoglobin, and OP_FG represents osteoporosis with pathological fractures.

In the LDSC analysis of five GWAS traits (hepatomegaly, splenic volume, total cholesterol, hemoglobin, and osteoporosis with pathological fractures), the *Z*‐scores for heritability contributions consistently exceeded 2, indicating high‐quality GWAS data. Genetic covariance values between traits are detailed in Figure 2 and Table S5. During SEM, the genetic covariance matrix of these five GD1‐related traits demonstrated excellent fit with the empirical covariance matrix under a common factor model (CFI = 0.975, *χ* ^2^/df = 1.141; Table S3). Parameters for the latent factor representing GD1 (F1) and single‐variable structural equation models are presented in Table S4. These results provided support for a shared genetic signal captured by the genomic SEM framework for GD1‐related traits.

### 3.2. Stability Assessment of Genomic SEM for GD1 Under LDSC

Following LDSC computation, our genomic SEM framework for GD1 incorporated parameter controls that excluded 4,187,467 SNPs while retaining 979,163 valid SNPs. Among these SNPs, the mean *χ* ^2^ was 1.276 (max *χ* ^2^ = 913.763), with *λ* GC = 0.853. All heterogeneity tests passed quality thresholds, yielding a genome‐wide significance level of 32. The estimated *h* ^2^ was 0.0016 (SE = 0.0002), and the regression intercept was 0.5488 (SE = 0.0027). These results suggested that the model fit was acceptable overall and that the observed signal was more consistent with polygenic effects than with substantial inflation due to population stratification or other confounding factors. Although the SNP heritability of the latent GD1 factor was modest, this estimate reflects the shared common‐variant component of a derived GD1‐related latent construct rather than the full heritable basis of clinically diagnosed GD1. Therefore, the subsequent genome‐wide analyses should be interpreted as exploratory.

### 3.3. FUMA‐Based Genomic SEM Risk Locus Assessment for GD1

**Figure 3:**
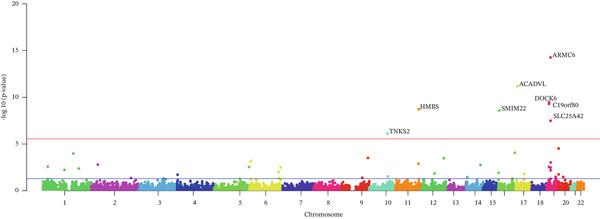
Manhattan plot of genes reaching the genome‐wide significance threshold.

**Figure 4:**
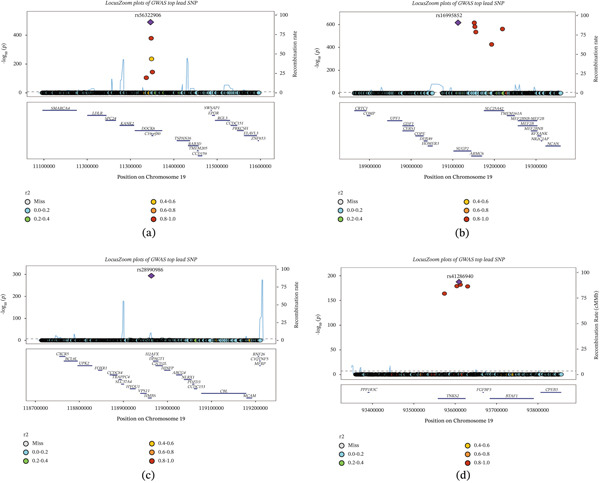
(a–d) LocusZoom plot of partial lead SNPs from the GWAS of GD1.

Next, we utilized FUMA software to evaluate risk loci within the genomic SEM framework for GD1. One hundred and sixty‐seven risk loci were identified (Table S6), with eight potential associated genes reaching the genome‐wide study threshold (sig.thres = 5e − 8 and FDR*p* < 0.05; Table S7, Figure 3). Through FUMA, 174 lead SNPs were detected, predominantly located in intergenic and intronic regions, with a minority in ncRNA intronic regions (Table S8, Figure 4). The GWAS‐by‐subtraction approach identified three SNPs: rs2293432, rs56322906, and rs112450640. rs2293432 has been reported in multiple studies to correlate with erythrocyte distribution width and very large VLDL, but it is not a direct causal variant for GD1, potentially representing a modifier locus. rs56322906 and rs112450640 show established associations with LDL cholesterol and Apolipoprotein B levels [22]; additionally, rs112450640 demonstrates links to Alzheimer′s disease [23], which aligns logically with the cognitive impairment phenotype observed in Gaucher disease.

### 3.4. Fine Mapping

**Figure 5:**
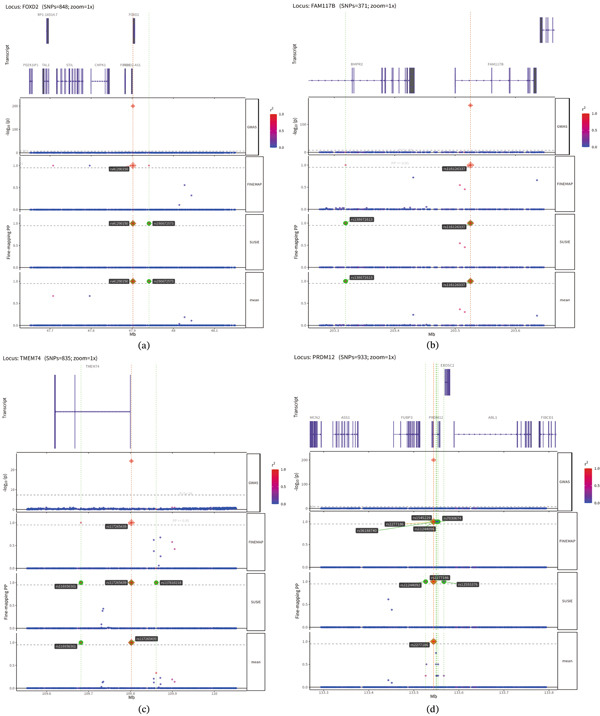
(a–d) Regional plot of partially strongly associated genomic regions reaching the mean.PP threshold in FINEMAP.

Through fine mapping analysis, we identified strong associations at 15 genomic loci, all exhibiting a mean posterior probability (mean.PP) exceeding 0.95. These included loci such as chr1:rs190672573 and rs41290150 (near *FOXD2*), chr2: rs116126337 and rs138672613 (near *FAM117B*), and chr8:rs116936361 and rs117265439 (near *TMEM74*), among others listed in Table S9. Regional plots demonstrated distinct peaks at these loci, with additional credible set variants providing supporting association evidence (Figure 5).

### 3.5. Identification of Susceptibility Genes for GD1

**Figure 6:**
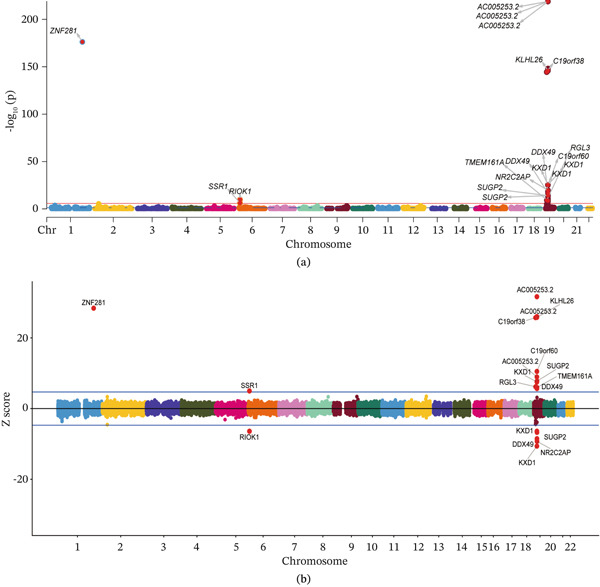
Manhattan plot of susceptibility genes identified by FUSION. (a) The *x*‐axis represents chromosome positions, the *y*‐axis represents the negative logarithm of *p* values, and the red line indicates the 5e − 8 threshold. (b) The *x*‐axis represents chromosome positions, and the *y*‐axis represents TWAS *Z*‐scores. The blue horizontal line in the figure represents the significance *Z*‐score of TWAS analysis, which is *Z* − score = ±1.96.

To identify susceptibility genes for GD1, we performed TWAS using FUSION to detect gene‐level associations related to GD1 molecular signatures. Nineteen genes surpassed the significance threshold of *p* < 5 × 10^−8^ in TWAS (Table S10, Figure 6). Subsequently, FOCUS was employed for fine mapping analysis, identifying 345 genes with PIP > 0.8, indicating their potential pathogenic roles. An intersection test was then conducted to prioritize high‐confidence gene‐level associations. By intersecting results from FUSION and FOCUS, 11 genes reached significance in both methods (Table 1). Among these, AC005253.2 emerged as the most strongly associated risk gene (*Z* − score = 31.63, *p* = 1.60e − 219), followed by ZNF281 (*Z* − score = 28.37, *p* = 5.46e − 177), suggesting that their elevated expression may confer GD1 risk. Conversely, RIOK1 was identified as the top protective gene (*Z* − score = −6.43, *p* = 1.30e − 10), with KXD1 ranking second (*Z* − score = −6.41, *p* = 1.46e − 10), indicating that reduced expression of these genes increases GD1 susceptibility.

**Table 1:** Susceptibility genes identified by FUSION and FOCUS.

ID	Chr	Start	End	TWAS.*Z*	TWAS.*p*	PIP
AC005253.2	19	18561518	18561519	31.63	1.60e − 219	1
ZNF281	1	200410055	200410056	28.37	5.46e − 177	1
AC005253.2	19	18561518	18561519	26	9.20e − 149	1
C19orf38	19	10836574	10836575	25.7	3.87e − 145	1
RIOK1	6	7389743	7389744	−6.43	1.30e − 10	1
KXD1	19	18557761	18557762	−6.41	1.46e − 10	0.98
DDX49	19	18919674	18919675	5.67	1.45e − 08	1
CCDC151	19	11435738	11435739	−3.4	6.66e − 04	1
DOCK6	19	11262451	11262452	−2.35	1.88e − 02	0.99
ADAM1B	12	111927017	111927018	2.13	3.32e − 02	1
CTC‐510F12.2	19	11203627	11203628	−2.08	3.78e − 02	0.98
SPC24	19	11155807	11155808	1.97	4.94e − 02	1

### 3.6. Molecular Dynamics Simulation and ddG Prediction

**Figure 7:**
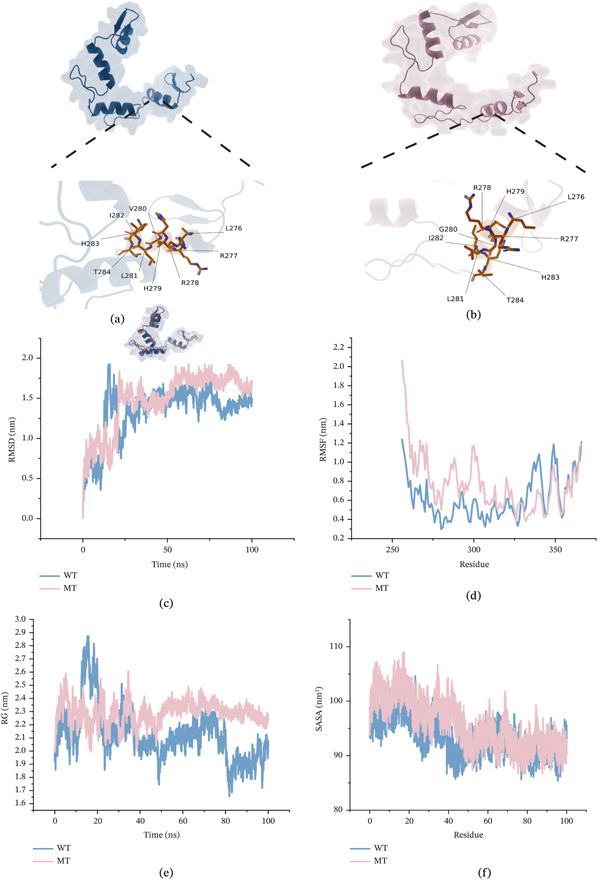
(a–f) Protein structure and dynamics simulation results of ZNF281.

**Figure 8:**
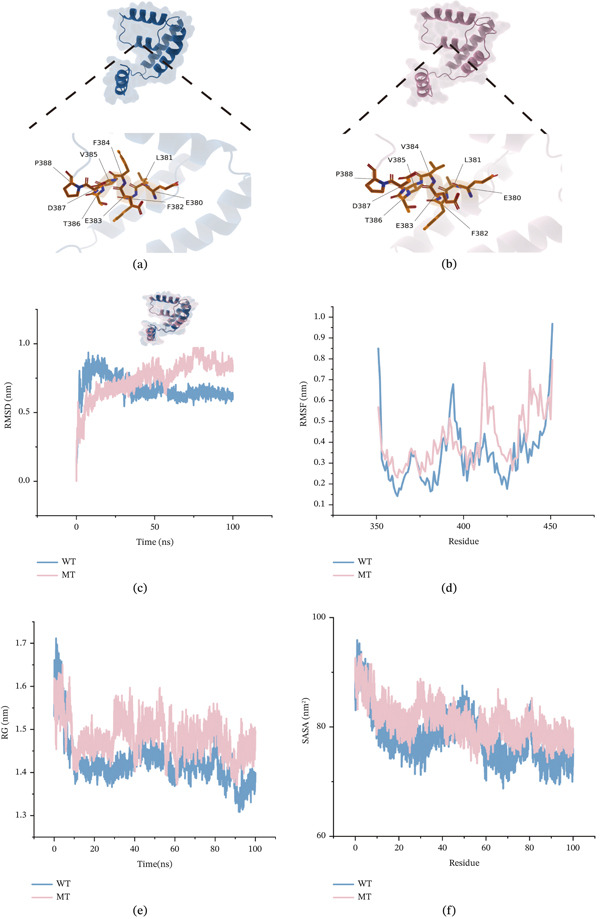
(a–f) Protein structure and dynamics simulation results of RIOK1.

ZNF281 and RIOK1 emerged as the most significant genes with positive and negative TWAS.*Z* scores, respectively. We obtained detailed variant information for both proteins (Table S11). Missense mutations resulted in amino acid substitutions at Position 280 (glycine [G]) in ZNF281 and Position 384 (valine [V]) in RIOK1. Using UniProt and AlphaFold3 modeling, we resolved WT and MT structures for both proteins (Figures 7a,b and 8a,b). Molecular dynamics simulations calculated four parameters: root mean square deviation (RMSD), root mean square fluctuation (RMSF), radius of gyration (Rg), and solvent accessible surface area (SASA). RMSD plots (Figures 7c and 8c) showed that WT structures maintained lower and relatively stable values (ZNF281: 1.25–1.5; RIOK1: 0.5–0.75), whereas MT structures exhibited larger fluctuations (ZNF281: 1.5–2; RIOK1: 0.625–1), suggesting decreased conformational stability after mutation. RMSF analysis (Figures 7d and 8d) showed increased flexibility in MT proteins at multiple residues, suggesting that the substitutions may have affected local interactions, including hydrogen bonding or hydrophobic interactions. Elevated Rg values in MTs (Figures 7e and 8e) suggested a relatively looser structural state, with a possible increase in the tendency toward misfolding. Increased SASA in MTs (Figures 7f and 8f) suggested greater exposure of the protein surface, which may affect intermolecular interactions.

**Figure 9:**
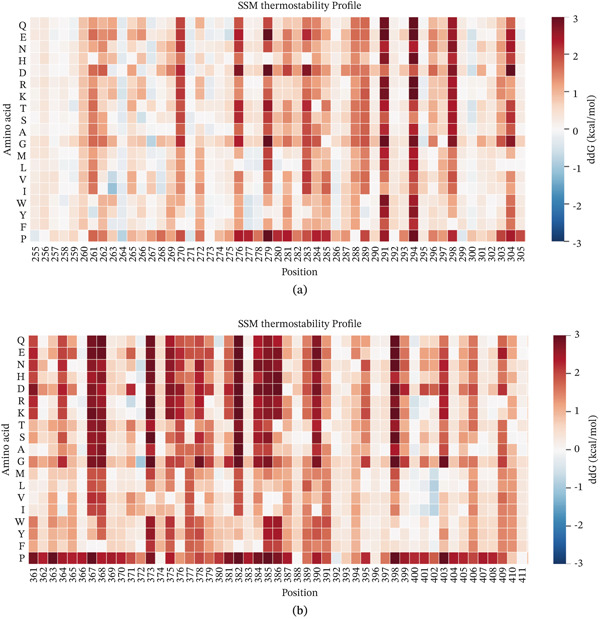
ddG prediction results of ZNF281 and RIOK1. (a) Position 280 is the mutation site of ZNF281, where the amino acid is substituted by glycine (G). (b) Position 384 is the mutation site of RIOK1, where the amino acid is substituted by valine (V).

The heat map in Figure 9 showed that, after amino acid substitution in ZNF281 and RIOK1, the corresponding positions shifted toward red, indicating an increase in ddG and reduced predicted structural stability. This finding was generally consistent with the molecular dynamics simulation results. Taken together, these results suggest that the selected missense variants may affect protein structure and stability. However, whether these changes translate into functional alterations relevant to GD1 still requires further experimental validation.

### 3.7. Gene Enrichment Analysis Based on MendelVar

**Figure 10:**
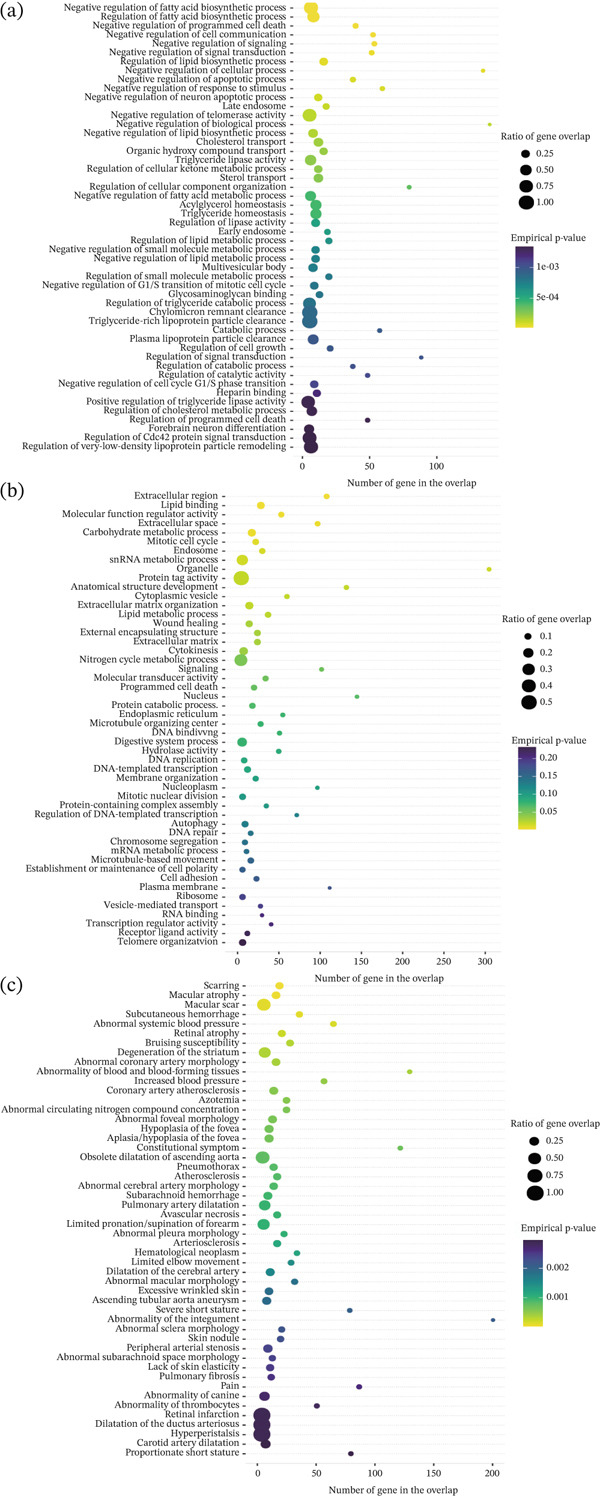
(a–c) Gene enrichment analysis results based on MendelVar.

Additionally, we employed the MendelVar tool to perform enrichment analysis on SNPs associated with GD1, covering biological processes, diseases, and other categories. Biological processes related to very‐low‐density lipoproteins, triglycerides, chylomicrons, neuronal regulation, cell cycle control, and signal transduction were significantly enriched (Figure 10a). These processes have been demonstrated to play crucial roles in the pathogenesis of GD1 [24–26]. Furthermore, functional terms closely linked to the disease—including hydrolase activity, protein catabolic process, autophagy, vesicle‐mediated transport, lipid metabolic process, and lipid binding—were also enriched (Figure 10b). In multisystem enrichment analysis, we successfully identified diseases co‐occurring with GD1 progression, such as platelet abnormalities, osteonecrosis, bone pain, pulmonary fibrosis, proportionate short stature, and retinal atrophy (Figure 10c). These results collectively provided additional biological context and were broadly consistent with pathways relevant to GD1‐related biology.

### 3.8. Biomarker and Risk Factor Identification

Next, we utilized the constructed GD1 GWAS as the outcome and GWAS data for 50,055 traits from the IEU database as exposures to identify biomarkers and related traits influencing the disease. Following FDR correction of results and filtering by heterogeneity test *p* values, we ultimately obtained 116 significant associations (Table S12). Among these, basal metabolic rate, whole body water mass, body mass index, low‐density lipoprotein, intermediate‐density lipoprotein, reticulocyte count, platelet distribution width, and aspartate aminotransferase were identified as risk factors for GD1. Conversely, monocyte count, white blood cell count, haptoglobin, total protein, mean alveolar volume, and systolic blood pressure were determined to be protective factors. Notably, while factors like low‐density lipoprotein, body weight, monocyte count, and mean alveolar volume had been previously associated with GD1 [27–29], novel factors, including intermediate‐density lipoprotein, total protein, and systolic blood pressure, emerged as biologically significant contributors, warranting further investigation.

### 3.9. PRSs Based on Summary Data

This study revealed that the PRS‐constructed variant loci were significantly associated with the risk of developing GD1, with distinct chromosomes demonstrating differential genetic contributions to the disease (Table S13). Specifically, Chromosome 1 exhibited the highest positive genetic contribution (score = 6.79e − 02), followed by Chromosome 21 (score = 4.35e − 02). Conversely, Chromosome 19 showed the strongest negative contribution (score = −1.97e − 01), with Chromosome 2 ranking second (score = −1.26e − 01). These findings indicated that these genomic regions likely harbored critical genes and regulatory elements that substantially influenced disease pathogenesis.

## 4. Discussion

GD1, a multisystem lysosomal storage disorder, presents complex comorbidities including progressive hepatosplenomegaly, bone disease, anemia, dyslipidemia, and pulmonary hypertension, posing significant clinical management challenges [3]. Although multisystem phenotypes often follow specific progression patterns, the genetic basis of comorbidities remains unclear. Traditional single‐trait GWASs face limitations in deciphering synergistic genetic mechanisms underlying multisystem damage in GD1 and fail to reveal phenotypic interaction networks [30]. Genomic SEM has been successfully applied to other complex diseases—such as elucidating etiological differences in bipolar disorder subtypes, identifying shared genetic risks across chronic pain conditions, and uncovering common genetic architecture among gastroesophageal reflux disease, asthma, and allergic disorders [31, 32]. This study integrated summary statistics from five GWAS datasets (hepatomegaly, splenic volume, total cholesterol, hemoglobin, and osteoporosis with pathological fractures). Employing a multimethod approach combining genomic SEM, summary data–based PRS, MR‐IEU, fine mapping, and transcriptomic analyses, we investigated shared genetic signals related to GD1 using an integrative analytical framework. Through joint analysis of these complex traits, several candidate genetic signals were highlighted. It should be noted that the present genomic SEM framework does not represent a direct GWAS of clinically diagnosed GD1 but rather a model of shared genetic liability across five GD1‐related traits.

Through LDSC analysis, this study revealed significant genetic covariance among the five incorporated GWAS traits, indicating shared genetic factors across these phenotypes with good model fit. Hepatomegaly, splenic volume, and total cholesterol emerged as major contributors, aligning with both the clinical manifestations of GD1 and the total cholesterol reduction reported in Le et al.′s study [33]. While hemoglobin and osteoporosis with pathological fractures showed relatively weaker GWAS effects compared to the first three traits, these phenotypes remain highly prevalent in GD1 progression [34, 35]. Our SEM further suggested that these traits may share overlapping genetic components rather than operating entirely independently.

Through genomic SEM and a series of GWAS analyses, three novel SNP loci were identified: rs2293432 (near HECTD4), rs56322906 (near DOCK6), and rs112450640 (near CBLC). These loci have not, to our knowledge, been highlighted previously in the context of the GD1‐related latent phenotype analyzed here. The rs2293432 variant is proximal to HECTD4, which encodes a HECT domain–containing protein involved in protein ubiquitination [36]. Ubiquitination plays a validated role in lysosomal degradation and autophagy—processes critical to GD1 pathogenesis [37, 38]. Furthermore, HECTD4 has been linked to neurodevelopmental disorders and epilepsy [39], reinforcing its relevance to GD1, which can manifest neurological symptoms despite not being a primary feature. The rs56322906 locus near DOCK6 (a Rho GTPase–activating protein) participates in cytoskeletal remodeling and cell migration [22, 40]. DOCK6 has been associated with lower plasma LDL‐C and HDL‐C levels [41], consistent with GD1 dyslipidemia, and reported in Paget′s disease of bone—characterized by bone destruction and pain that may contribute to GD1 skeletal pathology [42]. Crucially, Rho GTPases regulate macrophage processes including migration, morphological changes, and phagocytosis [43, 44], central to the formation of Gaucher cells in GD1 [45]. The rs112450640 variant near CBLC is implicated in intracellular signaling and endocytic pathways; CBLC maintains Golgi network integrity, essential for glucocerebrosidase metabolism—whose dysfunction underlies GD1 pathogenesis [46, 47].

Further fine mapping analysis identified 15 genomic loci surpassing the significant threshold. Although the genes near these loci have not been studied directly in GD1, previous work suggests that several of them may be biologically relevant to GD1‐related processes. ALDH1A2 has been repeatedly associated with osteoarthritis and neurological disorders [48]; LIPC links to familial hypocholesterolemia [49], aligning with GD1 comorbidities; FAM117B contributes to mitochondrial dysfunction and impaired autophagy [50], hindering lipid clearance in macrophages and exacerbating pathology; FOXD2 promotes chondrocyte survival while regulating macrophages and T cells [51, 52]; PIP4K2A modulates intracellular lipid trafficking and associates with anemia and depression—processes interconnected with GD1 [53–55]; PRDM12 is implicated in pain pathways [56], relevant to severe bone pain in GD1 patients; TMEM74, localized to lysosomes and autophagosomes, enhances autophagy under specific conditions [57], crucial for maintaining organelle function and clearing excess glucocerebroside in macrophages, with additional neurological disease associations [58].

Eleven genes surpassed the significance thresholds in both FUSION and FOCUS analyses, emerging as potential susceptibility genes for GD1. While research on AC005253.2 remains limited, its suppression has been shown to inhibit cellular proliferation and migration [59], suggesting that it may merit further investigation in the context of macrophage‐related processes relevant to GD1. KXD1, a BLOS1‐interacting protein and component of the BORC complex, participates in lysosome‐related organelle biogenesis and lysosomal trafficking [60]. Given GD1′s direct link to lysosomal dysfunction—where glucocerebroside accumulation requires functional lysosomal clearance—KXD1 therefore appears to be a biologically plausible candidate for further study. DDX49, an RNA helicase with ATPase activity, regulates preribosomal 47S RNA processing and global translational homeostasis [61], potentially influencing GD1 pathogenesis through ribosome biogenesis and glucocerebrosidase synthesis. SPC24, a microtubule‐associated spindle pole component, mediates mitotic spindle function [62] and contributes to ribosome biogenesis. Its dysfunction may affect cell‐cycle regulation and chromosome segregation, though its relevance to GD1 remains to be clarified [63]. We further examined selected missense variants in ZNF281 and RIOK1 because these genes showed prominent TWAS signals in opposite directions. The structural analyses suggested that the selected variants may affect protein conformation and stability, providing an additional layer of computational support for their prioritization. ZNF281 has been linked to liver injury, inflammation, and osteoporosis in previous studies [64, 65], whereas RIOK1 is involved in 40S ribosomal subunit maturation [66]. These observations suggest that both genes may be biologically relevant to GD1‐related processes, although the specific mechanisms remain uncertain. Notably, MendelVar‐based enrichment analysis provided additional biological context for the identified genes and was broadly consistent with pathways relevant to GD1‐related biology. Summary data PRS further supported the presence of heterogeneous chromosome‐level contributions to the GD1‐related latent trait [67]. Taking the negative contribution of Chromosome 19 as an example, this pattern is not necessarily indicative of a statistical artifact within the Bayesian regression model. Instead, it may indicate that this particular chromosomal region is highly enriched for protective alleles—specifically, variants with negative effect sizes—whose cumulative effect lowers the PRS for GD1 overall.

Our MR analysis highlighted several traits that showed putative associations with the GD1‐related latent phenotype. Basal metabolic rate, whole body water mass, body mass index, low‐density lipoprotein, intermediate‐density lipoprotein, reticulocyte count, platelet distribution width, and aspartate aminotransferase showed positive associations with the GD1‐related latent phenotype. Conversely, monocyte count, white blood cell count, haptoglobin, total protein, mean alveolar volume, and systolic blood pressure showed inverse associations. These findings may be useful for generating hypotheses regarding clinical correlates of GD1, but they should not yet be taken as a basis for direct monitoring recommendations. In terms of protective factors, taking total protein levels and systolic blood pressure as examples, higher total protein levels usually reflect better liver synthesis function (e.g., albumin synthesis) and sufficient nutritional status in the body, which may play a buffering and protective role in the severe consumptive course of late GD1. In addition, higher systolic blood pressure may reflect the patient′s retention of good vascular tone and autonomic nervous system stability, thereby antagonizing systemic vasodilation and dysfunction mediated by chronic macrophage inflammation. Notably, several factors represent replicated findings that reinforce their research significance in GD1 pathophysiology [68, 69].

While this study offers new insights into the genetic mechanisms of GD1, several limitations persist. Firstly, the sample in this study was primarily derived from European cohorts, which limits the generalizability of the findings to other populations. Furthermore, given the high carrier frequency of GD1, particularly the GBA1 N370S mutation, in the Ashkenazi Jewish population, future validation in Ashkenazi Jewish and other diverse ethnic groups is essential to ultimately confirm the clinical applicability of this genetic architecture model. Secondly, although multiple risk loci were identified through fine‐mapping and transcriptomics, their pathogenic mechanisms—including effects on gene expression, protein function, and metabolic pathways—remain uncharacterized, requiring future in‐depth investigation to refine the disease′s genetic landscape. We also acknowledge that the structural analyses reported here are computational in nature. At this stage, they are better understood as providing supportive clues rather than definitive mechanistic evidence. Additional experimental work, such as protein stability testing, cell‐based functional assays, and gene perturbation experiments, will be needed to clarify their biological relevance. Thirdly, beyond genetic factors, systematic exploration of environmental influences and their interplay with genes is essential to comprehensively elucidate phenotypic regulatory networks [68]. It should also be noted that the latent GD1 factor showed relatively low SNP heritability in the present analysis. Since this phenotype was reconstructed from several GD1‐related traits rather than directly measured, it probably reflects only the genetic component shared across those traits. This may have reduced the statistical strength of the subsequent analyses. For that reason, the loci and genes highlighted here should be viewed as preliminary and interpreted with appropriate caution until they are further examined in independent datasets, particularly in directly phenotyped GD1 cohorts.

## 5. Conclusions

Through genomic SEM and multidimensional post‐GWAS analyses, this study provides an initial characterization of shared genetic architecture related to GD1, identifying 174 significant lead SNPs—including three novel loci. Integration of FUSION and FOCUS further prioritized 11 candidate genes for future investigation. Protein dynamic simulations and ddG predictions suggested possible structural consequences of selected missense variants, while MR‐IEU analysis highlighted multiple traits that may be relevant to the GD1‐related latent phenotype.

## Author Contributions

Xiaomeng Lang and Jianping Liu: writing (review and editing) and conceptualization. Shijie Ren and Mingmin Du: writing (original draft), visualization, data curation, and methodology. Jingyuan Liu and Boyu Li: visualization, resources, and writing—original draft. All authors contributed to the paper. Shijie Ren and Mingmin Du are co‐first author.

## Funding

This study was funded by the Hebei Natural Science Foundation Project, H2022423326.

## Disclosure

All authors approved the submitted version

## Ethics Statement

The authors have nothing to report.

## Consent

The authors have nothing to report.

## Conflicts of Interest

The authors declare no conflicts of interest.

## Supporting information


**Supporting Information** Additional supporting information can be found online in the Supporting Information section. Table S1 GWAS summary sources. Table S2: SNP heritability of genomic SEM phenotypes. Table S3: Fit indices for genomic SEM model. Table S4: Genomic SEM factor loadings for Type 1 Gaucher disease. Table S5: Genetic correlations between Type 1 Gaucher disease. Table S6: Risk gene loci identified by FUMA. Table S7: Genes reaching genome‐wide significance threshold. Table S8: Lead SNPs identified by FUMA. Table S9: Results of FINEMAP analysis. Table S10: Significant genes identified by FUSION. Table S11: Detailed mutation information of ZNF281 and RIOK1. Table S12: Mendelian randomization results for 50,055 traits in the IEU database (FDR correction). Table S13: Polygenic risk score based on aggregated data.

## Data Availability

The GWAS data sources are listed in Table S1. FUMA can be accessed at https://fuma.ctglab.nl/, MendelVar is available at https://mendelvar.mrcieu.ac.uk/, Ensembl is hosted at https://www.ensembl.org, UniProt entries are found at https://www.uniprot.org/uniprotkb, and AlphaFold3 predictions are provided via https://alphafoldserver.com/. For further information, please contact the corresponding authors.
