# Supplementary material for: Genomic Structural Equation Modeling Provides an Initial View of the Genetic Architecture Related to Type 1 Gaucher Disease
Source: Hum Mutat. 2026 Apr 15;2026:1692822. doi: 10.1155/humu/1692822 (PMC13080874; doi:10.1155/humu/1692822)
Supplement: Supplementary file 1 — Supporting Information Additional supporting information can be found online in the Supporting Information section. Table S1 GWAS summary sources. Table S2: SNP heritability of genomic SEM phenotypes. Table S3: Fit indices for genomic SEM model. Table S4: Genomic SEM factor loadings for Type 1 Gaucher disease. Table S5: Genetic correlations between Type 1 Gaucher disease. Table S6: Risk gene loci identified by FUMA. Table S7: Genes reaching genome‐wide significance threshold. Table S8: Lead SNPs identified by FUMA. Table S9: Results of FINEMAP analysis. Table S10: Significant genes identified by FUSION. Table S11: Detailed mutation information of ZNF281 and RIOK1. Table S12: Mendelian randomization results for 50,055 traits in the IEU database (FDR correction). Table S13: Polygenic risk score based on aggregated data. [file HUMU-2026-1692822-s001.docx]

**Supplementary Table 1.** GWAS summary sources

| **Trait** | **Role in current study** | **Source** | **Download link** | **N case** | **N control** | **Total** |
| --- | --- | --- | --- | --- | --- | --- |
| Hepatomegaly | Genomic struture equation model | GCST90476092 | https://www.ebi.ac.uk/gwas/studies/GCST90476092 | 2861 | 441435 | 444296 |
| Spleen volume | Genomic struture equation model | GCST90016667 | https://www.ebi.ac.uk/gwas/studies/GCST90016667 | NA | NA | 32860 |
| Total cholesterol | Genomic struture equation model | IEU | https://gwas.mrcieu.ac.uk/datasets/met-d-Total_C/ | NA | NA | 115078 |
| Hemoglobin levels | Genomic struture equation model | GCST90426143 | https://www.ebi.ac.uk/gwas/studies/GCST90426143 | NA | NA | 3506 |
| Osteoporosis with pathological fracture (FG) | Genomic struture equation model | FinnGen R12 | https://storage.googleapis.com/finngen-public-data-r12/summary_stats/release/finngen_R12_OSTEOPOROSIS_FRACTURE_FG.gz | 2429 | 374317 | 376746 |
| IEU GWAS summary data | Mendelian randomization | IEU | https://gwas.mrcieu.ac.uk/datasets/ | - | - | - |

**Supplementary Table 2.** SNP heritability of Genomic-SEM phenotypes

| **Phenotype** | **NSNPs** | **h^2^ (se)** | **λGC** | **Mean ChiSquare** | **Intercept se** | **Ratio se** | **h^2^ Z** |
| --- | --- | --- | --- | --- | --- | --- | --- |
| Hepatomegaly | 1171864 | 0.0025 (0.0011) | 1.0374 | 1.0409 | 1.0195 (0.0066) | 0.477 (0.162) | 2.24 |
| Spleen volume | 1173079 | 0.2303 (0.0298) | 1.1474 | 1.1832 | 1.0301 (0.0088) | 0.1643 (0.0479) | 7.72 |
| Total cholesterol | 1175030 | 0.095 (0.0108) | 1.1474 | 1.2638 | 1.0427 (0.017) | 0.1618 (0.0644) | 8.76 |
| Hemoglobin levels | 1018382 | 0.1484 (0.1296) | 1.0125 | 1.0064 | 0.9962 (0.006) | 0.588 (0.9333) | 1.14 |
| Osteoporosis with pathological fracture (FG) | 1159740 | 0.0054 (0.0014) | 1.0702 | 1.0694 | 1.0295 (0.0078) | 0.4259 (0.1123) | 3.78 |

**Supplementary Table 3.** Fit Indices for Genomic-SEM Model

| **chisq** | **df** | **p_chisq** | **AIC** | **CFI** | **SRMR** |
| --- | --- | --- | --- | --- | --- |
| 5.705208 | 5 | 0.335968 | 25.70521 | 0.975485 | 0.132822 |

Note: chisq = Chi-Square Test Statistic, df = Degrees of Freedom, p_chisq = p-value for the Chi-Square Test, AIC = Akaike Information Criterion, CFI = Comparative Fit Index, SRMR = Standardized Root Mean Square Residual.

**Supplementary Table 4.** Genomic-SEM Factor Loadings for type 1 Gaucher disease

| **lhs** | **op** | **rhs** | **Unstandardized_Estimate** | **Unstandardized_SE** | **Standardized_Est** | **Standardized_SE** | **p_value** |
| --- | --- | --- | --- | --- | --- | --- | --- |
| F1 | =~ | Hepatomegaly | 0.106126 | 0.051764 | 0.367447 | 0.179225 | 0.040345 |
| F1 | =~ | Spleen volume | 0.412577 | 0.18219 | 0.859721 | 0.379644 | 0.023541 |
| F1 | =~ | Total cholesterol | -0.102878 | 0.045529 | 0.333842 | 0.147742 | 0.023844 |
| F1 | =~ | Hemoglobin levels | -0.11274 | 0.077206 | -0.29269 | 0.200443 | 0.144231 |
| F1 | =~ | Osteoporosis with FG | 0.039847 | 0.046715 | 0.091188 | 0.106906 | 0.393675 |

Abbreviations: lhs = Predictor, op = Operator ("=~" denotes factor loadings), rhs = Outcome, Unstandardized_Estimate = Unstandardized Factor Loading, Unstandardized_SE = Standard Error of the Unstandardized Estimate, Standardized_Est = Standardized Factor Loading, Standardized_SE = Standard Error of the Standardized Estimate.

**Supplementary Table 5.** Genetic correlations between type 1 Gaucher disease

|  | Hepatomegaly | Spleen volume | Total cholesterol | Hemoglobin levels | Osteoporosis with FG |
| --- | --- | --- | --- | --- | --- |
| Hepatomegaly | 0.0025 (0.0011) | 0.3074 (0.1300) | -0.1348 (0.1180) | 0.0437 (0.4463) | 0.1928 (0.2003) |
| Spleen volume | 0.3074 (0.1300) | 0.0025 (0.0011) | -0.2863 (0.0639) | -0.2224 (0.1846) | 0.1249 (0.0928) |
| Total cholesterol | -0.1348 (0.1180) | -0.2863 (0.0639) | 0.0025 (0.0011) | 0.2095 (0.1658) | 0.0664 (0.0804) |
| Hemoglobin levels | 0.0437 (0.4463) | -0.2224 (0.1846) | 0.2095 (0.1658) | 0.0025 (0.0011) | 0.4107 (0.3418) |
| Osteoporosis with FG | 0.1928 (0.2003) | 0.1249 (0.0928) | 0.0664 (0.0804) | 0.4107 (0.3418) | 0.0025 (0.0011) |

**Supplementary Table 6.** Risk gene loci identified by fuma

| **rsID** | **chr** | **p** | **start** | **end** | **nSNPs** | **nIndSigSNPs** | **IndSigSNPs** | **nLeadSNPs** | **LeadSNPs** |
| --- | --- | --- | --- | --- | --- | --- | --- | --- | --- |
| rs9439668 | 1 | 1.00E-200 | 18855886 | 18855974 | 2 | 1 | rs9439668 | 1 | rs9439668 |
| rs16829963 | 1 | 1.00E-200 | 25035348 | 25171862 | 13 | 1 | rs16829963 | 1 | rs16829963 |
| rs75470965 | 1 | 1.00E-200 | 38998839 | 39024203 | 14 | 1 | rs75470965 | 1 | rs75470965 |
| rs41290150 | 1 | 1.00E-200 | 47901586 | 47901586 | 1 | 1 | rs41290150 | 1 | rs41290150 |
| rs138457413 | 1 | 3.03E-11 | 49799284 | 49846498 | 3 | 1 | rs138457413 | 1 | rs138457413 |
| rs12084232 | 1 | 1.00E-200 | 85710249 | 85753812 | 14 | 1 | rs12084232 | 1 | rs12084232 |
| rs115485951 | 1 | 6.50E-09 | 107325728 | 107325728 | 1 | 1 | rs115485951 | 1 | rs115485951 |
| rs41281368 | 1 | 2.83E-15 | 110993967 | 110993967 | 1 | 1 | rs41281368 | 1 | rs41281368 |
| rs12140486 | 1 | 3.47E-17 | 156820294 | 156820814 | 2 | 1 | rs12140486 | 1 | rs12140486 |
| rs79091515 | 1 | 3.18E-31 | 185026908 | 185026908 | 1 | 1 | rs79091515 | 1 | rs79091515 |
| rs12120143 | 1 | 1.00E-200 | 197438417 | 197499003 | 2 | 1 | rs12120143 | 1 | rs12120143 |
| rs12070749 | 1 | 1.00E-200 | 200670438 | 200857445 | 76 | 1 | rs12070749 | 1 | rs12070749 |
| rs72736897 | 1 | 1.00E-200 | 218406739 | 218406739 | 1 | 1 | rs72736897 | 1 | rs72736897 |
| rs115823518 | 1 | 3.38E-29 | 237901816 | 237901816 | 1 | 1 | rs115823518 | 1 | rs115823518 |
| rs79005908 | 1 | 7.38E-34 | 239041979 | 239117209 | 10 | 1 | rs79005908 | 1 | rs79005908 |
| rs60485084 | 2 | 1.00E-200 | 33210538 | 33315242 | 22 | 1 | rs60485084 | 1 | rs60485084 |
| rs72789423 | 2 | 1.00E-200 | 41231193 | 41259315 | 4 | 1 | rs72789423 | 1 | rs72789423 |
| rs10153553 | 2 | 1.00E-200 | 72038203 | 72091275 | 18 | 1 | rs10153553 | 1 | rs10153553 |
| rs142629349 | 2 | 4.48E-51 | 105808452 | 105808452 | 1 | 1 | rs142629349 | 1 | rs142629349 |
| rs61636499 | 2 | 5.99E-12 | 116775371 | 116861824 | 34 | 1 | rs61636499 | 1 | rs61636499 |
| rs72834353 | 2 | 1.00E-200 | 118507677 | 118842801 | 78 | 1 | rs72834353 | 1 | rs72834353 |
| rs2675078 | 2 | 1.00E-200 | 183711970 | 183981944 | 36 | 1 | rs2675078 | 1 | rs2675078 |
| rs113695308 | 2 | 1.00E-200 | 190112828 | 190282000 | 5 | 1 | rs113695308 | 1 | rs113695308 |
| rs4356612 | 2 | 1.00E-200 | 192796832 | 192815883 | 4 | 1 | rs4356612 | 1 | rs4356612 |
| rs116126337 | 2 | 4.38E-169 | 203047054 | 203525422 | 4 | 1 | rs116126337 | 1 | rs116126337 |
| rs76639598 | 3 | 1.00E-200 | 5800497 | 5800497 | 1 | 1 | rs76639598 | 1 | rs76639598 |
| rs77558416 | 3 | 5.60E-120 | 31905786 | 31905786 | 1 | 1 | rs77558416 | 1 | rs77558416 |
| rs4676489 | 3 | 6.75E-17 | 39313524 | 39402367 | 15 | 1 | rs4676489 | 1 | rs4676489 |
| rs376612740 | 3 | 1.92E-189 | 112379746 | 112382391 | 2 | 1 | rs376612740 | 1 | rs376612740 |
| rs10935335 | 3 | 1.00E-200 | 139294272 | 139294272 | 1 | 1 | rs10935335 | 1 | rs10935335 |
| rs76083848 | 3 | 1.00E-200 | 144933634 | 144933634 | 1 | 1 | rs76083848 | 1 | rs76083848 |
| rs114775298 | 3 | 3.79E-21 | 168457596 | 168503010 | 3 | 1 | rs114775298 | 1 | rs114775298 |
| rs62289859 | 3 | 1.00E-200 | 184880466 | 184906201 | 10 | 1 | rs62289859 | 1 | rs62289859 |
| rs139543189 | 3 | 1.21E-93 | 197196403 | 197233346 | 2 | 1 | rs139543189 | 1 | rs139543189 |
| rs75807625 | 4 | 4.00E-200 | 2561262 | 2735108 | 22 | 1 | rs75807625 | 1 | rs75807625 |
| rs61756977 | 4 | 1.37E-17 | 47802162 | 48035527 | 2 | 1 | rs61756977 | 1 | rs61756977 |
| rs150957872 | 4 | 1.32E-29 | 72081733 | 72266729 | 3 | 1 | rs150957872 | 1 | rs150957872 |
| rs76870542 | 4 | 3.09E-60 | 90612588 | 90619950 | 2 | 1 | rs76870542 | 1 | rs76870542 |
| rs112201606 | 4 | 1.00E-200 | 92382457 | 92612240 | 2 | 1 | rs112201606 | 1 | rs112201606 |
| rs74332078 | 4 | 1.00E-200 | 103685218 | 103949607 | 3 | 1 | rs74332078 | 1 | rs74332078 |
| rs13137555 | 4 | 6.72E-27 | 111133509 | 111246762 | 16 | 1 | rs13137555 | 1 | rs13137555 |
| rs74701742 | 4 | 1.00E-200 | 121503549 | 121525128 | 5 | 1 | rs74701742 | 1 | rs74701742 |
| rs115888840 | 4 | 1.27E-10 | 173135565 | 173188720 | 3 | 1 | rs115888840 | 1 | rs115888840 |
| rs74534063 | 4 | 7.42E-200 | 182945244 | 182958782 | 4 | 1 | rs74534063 | 1 | rs74534063 |
| rs72730669 | 5 | 1.00E-200 | 37931502 | 37992631 | 19 | 1 | rs72730669 | 1 | rs72730669 |
| rs2337414 | 5 | 1.00E-200 | 44970564 | 45885151 | 6 | 1 | rs2337414 | 1 | rs2337414 |
| rs16878633 | 5 | 8.79E-10 | 59861408 | 60583560 | 44 | 1 | rs16878633 | 1 | rs16878633 |
| rs76958242 | 5 | 1.00E-200 | 75009084 | 75121644 | 26 | 1 | rs76958242 | 1 | rs76958242 |
| rs16902844 | 5 | 1.00E-200 | 87143483 | 87143483 | 1 | 1 | rs16902844 | 1 | rs16902844 |
| rs112112085 | 5 | 5.28E-20 | 104082420 | 104085856 | 2 | 1 | rs112112085 | 1 | rs112112085 |
| rs10067453 | 5 | 2.55E-30 | 111863431 | 111933447 | 5 | 1 | rs10067453 | 1 | rs10067453 |
| rs77716125 | 5 | 1.72E-133 | 141929795 | 141961747 | 11 | 1 | rs77716125 | 1 | rs77716125 |
| rs114875219 | 5 | 1.67E-41 | 147442210 | 147541615 | 17 | 1 | rs114875219 | 1 | rs114875219 |
| rs116570776 | 5 | 1.06E-128 | 156835085 | 156835085 | 1 | 1 | rs116570776 | 1 | rs116570776 |
| rs11743318 | 5 | 1.00E-200 | 177605805 | 177622284 | 7 | 1 | rs11743318 | 1 | rs11743318 |
| rs17078961 | 5 | 6.29E-194 | 178424238 | 178528838 | 14 | 1 | rs17078961 | 1 | rs17078961 |
| rs74781311 | 6 | 1.37E-17 | 6839193 | 6858242 | 3 | 1 | rs74781311 | 1 | rs74781311 |
| rs9392879 | 6 | 1.11E-71 | 7345283 | 7345283 | 1 | 1 | rs9392879 | 1 | rs9392879 |
| rs17398435 | 6 | 1.00E-200 | 7604106 | 7604106 | 1 | 1 | rs17398435 | 1 | rs17398435 |
| rs62389223 | 6 | 1.13E-189 | 12779759 | 12860987 | 10 | 1 | rs62389223 | 1 | rs62389223 |
| rs71554596 | 6 | 1.00E-200 | 16993012 | 17010141 | 2 | 1 | rs71554596 | 1 | rs71554596 |
| rs7452568 | 6 | 1.00E-200 | 23592495 | 23593581 | 2 | 1 | rs7452568 | 1 | rs7452568 |
| rs7774931 | 6 | 1.00E-200 | 54156398 | 54159874 | 2 | 1 | rs7774931 | 1 | rs7774931 |
| rs141908879 | 6 | 1.35E-109 | 66025248 | 66025248 | 1 | 1 | rs141908879 | 1 | rs141908879 |
| rs143468892 | 6 | 1.00E-200 | 99989916 | 100089595 | 3 | 1 | rs143468892 | 1 | rs143468892 |
| rs113154201 | 6 | 6.14E-12 | 107298354 | 107298354 | 1 | 1 | rs113154201 | 1 | rs113154201 |
| rs62439706 | 6 | 1.00E-200 | 150179380 | 150179380 | 1 | 1 | rs62439706 | 1 | rs62439706 |
| rs2128948 | 6 | 4.25E-67 | 151655932 | 151667030 | 6 | 1 | rs2128948 | 1 | rs2128948 |
| rs7761031 | 6 | 1.00E-200 | 160654622 | 160758982 | 21 | 1 | rs7761031 | 1 | rs7761031 |
| rs78108244 | 6 | 3.26E-26 | 165385599 | 165389255 | 2 | 1 | rs78108244 | 1 | rs78108244 |
| rs35676495 | 7 | 1.00E-200 | 1144767 | 1150899 | 2 | 1 | rs35676495 | 1 | rs35676495 |
| rs112772005 | 7 | 8.81E-17 | 21528655 | 21654653 | 2 | 1 | rs112772005 | 1 | rs112772005 |
| rs117481973 | 7 | 7.08E-33 | 105841082 | 105904130 | 2 | 1 | rs117481973 | 1 | rs117481973 |
| rs191056729 | 7 | 1.71E-13 | 119682633 | 119775240 | 97 | 1 | rs191056729 | 1 | rs191056729 |
| rs117061478 | 7 | 1.09E-09 | 123870149 | 124066753 | 30 | 1 | rs117061478 | 1 | rs117061478 |
| rs56317486 | 7 | 3.32E-189 | 124957670 | 125092082 | 19 | 1 | rs56317486 | 1 | rs56317486 |
| rs17867320 | 7 | 1.00E-200 | 126848854 | 126864528 | 8 | 1 | rs17867320 | 1 | rs17867320 |
| rs114504887 | 8 | 3.17E-51 | 3713666 | 3761444 | 18 | 1 | rs114504887 | 1 | rs114504887 |
| rs17717355 | 8 | 1.00E-200 | 9197722 | 9198695 | 2 | 1 | rs17717355 | 1 | rs17717355 |
| rs78524955 | 8 | 1.00E-200 | 18439844 | 18464910 | 20 | 1 | rs78524955 | 1 | rs78524955 |
| rs36039502 | 8 | 1.00E-200 | 21002414 | 21002414 | 1 | 1 | rs36039502 | 1 | rs36039502 |
| rs138859866 | 8 | 2.75E-96 | 23759814 | 24261547 | 9 | 1 | rs138859866 | 1 | rs138859866 |
| rs72643032 | 8 | 1.00E-200 | 37808901 | 37873503 | 30 | 1 | rs72643032 | 1 | rs72643032 |
| rs192536583 | 8 | 1.00E-200 | 65852335 | 65965728 | 3 | 1 | rs192536583 | 1 | rs192536583 |
| rs28756805 | 8 | 1.00E-200 | 95845270 | 95847357 | 2 | 1 | rs28756805 | 1 | rs28756805 |
| rs117265439 | 8 | 4.04E-25 | 109756222 | 109802217 | 2 | 1 | rs117265439 | 1 | rs117265439 |
| rs17729371 | 8 | 1.00E-200 | 116708602 | 116744022 | 2 | 1 | rs17729371 | 1 | rs17729371 |
| rs35705938 | 8 | 1.00E-200 | 120134308 | 120134308 | 1 | 1 | rs35705938 | 1 | rs35705938 |
| rs1597280 | 8 | 1.00E-200 | 134762954 | 134775266 | 3 | 1 | rs1597280 | 1 | rs1597280 |
| rs117719340 | 9 | 7.39E-121 | 30887074 | 30887074 | 1 | 1 | rs117719340 | 1 | rs117719340 |
| rs149926044 | 9 | 1.00E-200 | 87587550 | 87587550 | 1 | 1 | rs149926044 | 1 | rs149926044 |
| rs72747427 | 9 | 1.00E-200 | 96413594 | 96413594 | 1 | 1 | rs72747427 | 1 | rs72747427 |
| rs11787891 | 9 | 1.00E-200 | 101675724 | 101680380 | 5 | 1 | rs11787891 | 1 | rs11787891 |
| rs2026362 | 9 | 1.00E-200 | 104230308 | 104240754 | 6 | 1 | rs2026362 | 1 | rs2026362 |
| rs62574705 | 9 | 1.00E-200 | 121329339 | 121357574 | 17 | 1 | rs62574705 | 1 | rs62574705 |
| rs68184458 | 9 | 1.00E-200 | 129288515 | 129303816 | 10 | 1 | rs68184458 | 1 | rs68184458 |
| rs2277186 | 9 | 1.00E-200 | 133537243 | 133543981 | 3 | 1 | rs2277186 | 1 | rs2277186 |
| rs147278971 | 9 | 6.40E-61 | 135956068 | 136174495 | 4 | 2 | rs192129994;rs147278971 | 2 | rs192129994;rs147278971 |
| rs12779592 | 10 | 2.17E-11 | 2378293 | 2413594 | 11 | 1 | rs12779592 | 1 | rs12779592 |
| rs9787428 | 10 | 1.00E-200 | 22809255 | 22854767 | 10 | 1 | rs9787428 | 1 | rs9787428 |
| rs150489002 | 10 | 1.22E-151 | 26062837 | 26095093 | 2 | 1 | rs150489002 | 1 | rs150489002 |
| rs4745982 | 10 | 1.00E-200 | 71089843 | 71089843 | 1 | 1 | rs4745982 | 1 | rs4745982 |
| rs111532642 | 10 | 9.14E-19 | 81087346 | 81143553 | 55 | 1 | rs111532642 | 1 | rs111532642 |
| rs61854881 | 10 | 9.60E-110 | 90878904 | 90878904 | 1 | 1 | rs61854881 | 1 | rs61854881 |
| rs41286940 | 10 | 1.87E-188 | 93442801 | 93650630 | 71 | 1 | rs41286940 | 1 | rs41286940 |
| rs61736835 | 10 | 1.00E-200 | 95367068 | 95657377 | 11 | 2 | rs56187102;rs61736835 | 2 | rs56187102;rs61736835 |
| rs183518579 | 10 | 3.31E-32 | 100466392 | 100862091 | 9 | 1 | rs183518579 | 1 | rs183518579 |
| rs78491197 | 10 | 7.70E-64 | 117898401 | 117954139 | 10 | 1 | rs78491197 | 1 | rs78491197 |
| rs117973923 | 11 | 1.78E-85 | 25311104 | 25369481 | 6 | 1 | rs117973923 | 1 | rs117973923 |
| rs11037543 | 11 | 1.00E-200 | 43611811 | 43744158 | 3 | 1 | rs11037543 | 1 | rs11037543 |
| rs79999706 | 11 | 1.00E-200 | 96748968 | 96771009 | 4 | 1 | rs79999706 | 1 | rs79999706 |
| rs3016356 | 11 | 1.00E-200 | 116477772 | 117095283 | 122 | 4 | rs3016356;rs75542613;rs1135663;rs59781045 | 3 | rs3016356;rs75542613;rs59781045 |
| rs28990986 | 11 | 1.00E-200 | 118953952 | 118963291 | 2 | 1 | rs28990986 | 1 | rs28990986 |
| rs367998292 | 11 | 1.00E-200 | 121670712 | 121735009 | 9 | 2 | rs367998292;rs12280388 | 1 | rs367998292 |
| rs145765147 | 12 | 2.21E-23 | 7891492 | 7891492 | 1 | 1 | rs145765147 | 1 | rs145765147 |
| rs56058261 | 12 | 5.90E-58 | 12544111 | 12544111 | 1 | 1 | rs56058261 | 1 | rs56058261 |
| rs74588265 | 12 | 1.10E-95 | 20989232 | 21306839 | 7 | 1 | rs74588265 | 1 | rs74588265 |
| rs73088639 | 12 | 2.08E-25 | 51312457 | 51572347 | 5 | 1 | rs73088639 | 1 | rs73088639 |
| rs61924767 | 12 | 1.94E-194 | 65504149 | 65504149 | 1 | 1 | rs61924767 | 1 | rs61924767 |
| rs117178443 | 12 | 1.00E-200 | 76689666 | 77122002 | 6 | 2 | rs144814086;rs117178443 | 2 | rs144814086;rs117178443 |
| rs79168361 | 12 | 2.59E-13 | 97167398 | 97414698 | 44 | 1 | rs79168361 | 1 | rs79168361 |
| rs146929492 | 12 | 1.75E-18 | 100472208 | 100742150 | 3 | 1 | rs146929492 | 1 | rs146929492 |
| rs2293432 | 12 | 1.00E-200 | 111883779 | 113018479 | 206 | 1 | rs2293432 | 1 | rs2293432 |
| rs142268019 | 13 | 9.24E-26 | 70771892 | 70784181 | 2 | 1 | rs142268019 | 1 | rs142268019 |
| rs7318349 | 13 | 1.27E-197 | 72966066 | 72976336 | 4 | 1 | rs7318349 | 1 | rs7318349 |
| rs17110331 | 14 | 1.00E-200 | 26188090 | 26277677 | 25 | 1 | rs17110331 | 1 | rs17110331 |
| rs140258097 | 14 | 2.01E-22 | 82217909 | 82250557 | 2 | 1 | rs140258097 | 1 | rs140258097 |
| rs78430956 | 14 | 2.53E-30 | 92210640 | 92332949 | 57 | 1 | rs78430956 | 1 | rs78430956 |
| rs79881262 | 14 | 1.00E-200 | 98689053 | 98750119 | 15 | 1 | rs79881262 | 1 | rs79881262 |
| rs145691643 | 15 | 1.00E-200 | 48796978 | 48812477 | 2 | 1 | rs145691643 | 1 | rs145691643 |
| rs4646583 | 15 | 1.00E-200 | 58097304 | 58353810 | 46 | 2 | rs138380023;rs4646583 | 2 | rs138380023;rs4646583 |
| rs77027049 | 15 | 1.00E-200 | 58716377 | 58716647 | 2 | 1 | rs77027049 | 1 | rs77027049 |
| rs4776293 | 15 | 1.00E-200 | 66528324 | 66703629 | 7 | 1 | rs4776293 | 1 | rs4776293 |
| rs8048774 | 16 | 1.61E-128 | 1358496 | 1530948 | 20 | 1 | rs8048774 | 1 | rs8048774 |
| rs145940844 | 16 | 1.00E-200 | 4817568 | 4952614 | 4 | 1 | rs145940844 | 1 | rs145940844 |
| rs78763534 | 16 | 1.00E-200 | 6088593 | 6109657 | 5 | 1 | rs78763534 | 1 | rs78763534 |
| rs111837854 | 16 | 1.00E-200 | 7014739 | 7041673 | 3 | 1 | rs111837854 | 1 | rs111837854 |
| rs79322314 | 16 | 1.00E-200 | 74883956 | 74883956 | 1 | 1 | rs79322314 | 1 | rs79322314 |
| rs28379208 | 16 | 4.46E-92 | 84000437 | 84035196 | 41 | 1 | rs28379208 | 1 | rs28379208 |
| rs9940232 | 16 | 3.36E-103 | 87425696 | 87584974 | 3 | 1 | rs9940232 | 1 | rs9940232 |
| rs77680021 | 17 | 3.90E-200 | 7124506 | 7124506 | 1 | 1 | rs77680021 | 1 | rs77680021 |
| rs76868109 | 17 | 1.00E-200 | 41776943 | 41874745 | 2 | 1 | rs76868109 | 1 | rs76868109 |
| rs113741838 | 17 | 1.00E-200 | 49029897 | 49193209 | 11 | 1 | rs113741838 | 1 | rs113741838 |
| rs17700212 | 17 | 4.60E-199 | 65107857 | 65232692 | 3 | 1 | rs17700212 | 1 | rs17700212 |
| rs77293315 | 18 | 3.85E-48 | 24702245 | 24702245 | 1 | 1 | rs77293315 | 1 | rs77293315 |
| rs146815093 | 18 | 1.29E-09 | 26654035 | 26654035 | 1 | 1 | rs146815093 | 1 | rs146815093 |
| rs17068330 | 18 | 1.00E-200 | 59122933 | 59142259 | 20 | 1 | rs17068330 | 1 | rs17068330 |
| rs75152146 | 18 | 1.27E-18 | 60437638 | 60600895 | 3 | 1 | rs75152146 | 1 | rs75152146 |
| rs150172415 | 18 | 1.89E-29 | 62125113 | 62125113 | 1 | 1 | rs150172415 | 1 | rs150172415 |
| rs117831057 | 19 | 1.00E-200 | 10694720 | 10707853 | 2 | 1 | rs117831057 | 1 | rs117831057 |
| rs56322906 | 19 | 1.00E-200 | 11311885 | 11350488 | 18 | 2 | rs56322906;rs3745683 | 1 | rs56322906 |
| rs16995852 | 19 | 1.00E-200 | 19108587 | 19246680 | 10 | 1 | rs16995852 | 1 | rs16995852 |
| rs117414940 | 19 | 4.55E-37 | 19855549 | 19855549 | 1 | 1 | rs117414940 | 1 | rs117414940 |
| rs62116889 | 19 | 2.75E-20 | 45022560 | 45033485 | 4 | 1 | rs62116889 | 1 | rs62116889 |
| rs112450640 | 19 | 1.78E-14 | 45296364 | 45296364 | 1 | 1 | rs112450640 | 1 | rs112450640 |
| rs117803469 | 20 | 1.12E-39 | 1600405 | 1600405 | 1 | 1 | rs117803469 | 1 | rs117803469 |
| rs56219025 | 20 | 1.00E-200 | 25239801 | 25756477 | 4 | 1 | rs56219025 | 1 | rs56219025 |
| rs80304109 | 20 | 4.10E-27 | 32737601 | 32739552 | 2 | 1 | rs80304109 | 1 | rs80304109 |
| rs79906004 | 20 | 2.33E-18 | 35697933 | 35854324 | 2 | 1 | rs79906004 | 1 | rs79906004 |
| rs117162746 | 20 | 1.00E-200 | 38669467 | 38719572 | 14 | 1 | rs117162746 | 1 | rs117162746 |
| rs6130478 | 20 | 1.14E-08 | 42533655 | 42533655 | 1 | 1 | rs6130478 | 1 | rs6130478 |
| rs6121704 | 20 | 1.00E-200 | 60144274 | 60170767 | 28 | 1 | rs6121704 | 1 | rs6121704 |
| rs75978011 | 21 | 1.00E-200 | 22954930 | 23268144 | 11 | 2 | rs75978011;rs73229346 | 2 | rs75978011;rs73229346 |
| rs73905204 | 21 | 1.00E-200 | 36604770 | 36617966 | 6 | 1 | rs73905204 | 1 | rs73905204 |
| rs12053781 | 22 | 1.00E-200 | 26581926 | 26581926 | 1 | 1 | rs12053781 | 1 | rs12053781 |
| rs116923341 | 22 | 4.83E-91 | 35259690 | 35259690 | 1 | 1 | rs116923341 | 1 | rs116923341 |
| rs73179581 | 22 | 1.00E-200 | 49290633 | 49311845 | 5 | 1 | rs73179581 | 1 | rs73179581 |

**Supplementary Table 7.** Genes reaching genome-wide significance threshold

| **GENE** | **CHR** | **START** | **STOP** | **N** | **ZSTAT** | **P** | **SYMBOL** | **FDR** |
| --- | --- | --- | --- | --- | --- | --- | --- | --- |
| ENSG00000107854 | 10 | 93558069 | 93625033 | 1781499 | 4.8098 | 7.5539e-07 | TNKS2 | 0.00171 |
| ENSG00000256269 | 11 | 118955576 | 118964259 | 1781499 | 5.8952 | 1.8712e-09 | HMBS | 6.76289104e-06 |
| ENSG00000267795 | 16 | 4838398 | 4846492 | 1781499 | 5.8405 | 2.6022e-09 | SMIM22 | 7.8373927e-06 |
| ENSG00000072778 | 17 | 7120444 | 7128592 | 1781499 | 6.767 | 6.5744e-12 | ACADVL | 5.94029912e-08 |
| ENSG00000130158 | 19 | 11309971 | 11373157 | 1781499 | 6.1681 | 3.4549e-10 | DOCK6 | 2.08111659666667e-06 |
| ENSG00000130173 | 19 | 11348178 | 11352619 | 1781499 | 6.1094 | 5e-10 | C19orf80 | 2.258875e-06 |
| ENSG00000105676 | 19 | 19144384 | 19170563 | 1781499 | 7.7272 | 5.4956e-15 | ARMC6 | 9.93109876e-11 |
| ENSG00000181035 | 19 | 19174808 | 19223697 | 1781499 | 5.4048 | 3.2435e-08 | SLC25A42 | 8.37332692857143e-05 |

**Supplementary Table 8.** Lead SNPs identified by FUMA

| **rsID** | **CHR** | **BP** | **IndSigSNPs** | **MAF** | **p** | **nearestGene** | **func** | **CADD** |
| --- | --- | --- | --- | --- | --- | --- | --- | --- |
| rs9439668 | 1 | 18855974 | rs9439668 | 0.037773 | 1e-200 | RP1-8B22.1 | intergenic | 0.351 |
| rs16829963 | 1 | 25044726 | rs16829963 | 0.036779 | 1e-200 | CLIC4 | intergenic | 0.586 |
| rs75470965 | 1 | 39014639 | rs75470965 | 0.070577 | 1e-200 | RP11-329N22.1 | intergenic | 0.754 |
| rs41290150 | 1 | 47901586 | rs41290150 | 0.012923 | 1e-200 | FOXD2 | upstream | 4.841 |
| rs138457413 | 1 | 49799284 | rs138457413 | 0.014911 | 3.02984606174e-11 | AGBL4 | intronic | 0.635 |
| rs12084232 | 1 | 85750571 | rs12084232 | 0.076541 | 1e-200 | RP11-131L23.1 | ncRNA_intronic | 5.517 |
| rs115485951 | 1 | 107325728 | rs115485951 | 0.025845 | 6.50065722645e-09 | RP11-110F24.1 | intergenic | 0.763 |
| rs41281368 | 1 | 110993967 | rs41281368 | 0.026839 | 2.82773844097e-15 | PROK1 | intronic | 0.113 |
| rs12140486 | 1 | 156820814 | rs12140486 | 0.038767 | 3.47107765366e-17 | NTRK1:INSRR | intronic | 10.93 |
| rs79091515 | 1 | 185026908 | rs79091515 | 0.024851 | 3.17645406292e-31 | RNF2 | intronic | 0.469 |
| rs12120143 | 1 | 197499003 | rs12120143 | 0.017893 | 1e-200 | DENND1B | intronic | 11.57 |
| rs12070749 | 1 | 200704310 | rs12070749 | 0.097416 | 1e-200 | CAMSAP2 | intergenic | 12.26 |
| rs72736897 | 1 | 218406739 | rs72736897 | 0.070577 | 1e-200 | RRP15 | intergenic | 1.409 |
| rs115823518 | 1 | 237901816 | rs115823518 | 0.026839 | 3.3789743826e-29 | RYR2 | intronic | 3.194 |
| rs79005908 | 1 | 239108744 | rs79005908 | 0.023857 | 7.3773988739e-34 | RP11-307O1.1 | intergenic | 0.359 |
| rs60485084 | 2 | 33218729 | rs60485084 | 0.073559 | 1e-200 | LTBP1 | intronic | 3.544 |
| rs72789423 | 2 | 41231193 | rs72789423 | 0.070577 | 1e-200 | HNRNPA1P57 | intergenic | 9.247 |
| rs10153553 | 2 | 72056774 | rs10153553 | 0.077535 | 1e-200 | DYSF | intergenic | 14.11 |
| rs142629349 | 2 | 105808452 | rs142629349 | 0.022863 | 4.48445846495e-51 | AC104655.3 | intergenic | 10.7 |
| rs61636499 | 2 | 116830021 | rs61636499 | 0.173956 | 5.99036140226e-12 | DPP10 | intergenic | 2.498 |
| rs72834353 | 2 | 118639163 | rs72834353 | 0.073559 | 1e-200 | HTR5BP | ncRNA_intronic | 1.813 |
| rs2675078 | 2 | 183923155 | rs2675078 | 0.071571 | 1e-200 | SNORA77 | intergenic | 1.127 |
| rs113695308 | 2 | 190282000 | rs113695308 | 0.040756 | 1e-200 | WDR75 | intergenic | 3.388 |
| rs4356612 | 2 | 192796832 | rs4356612 | 0.075547 | 1e-200 | AC098617.1 | ncRNA_intronic | 4.501 |
| rs116126337 | 2 | 203525422 | rs116126337 | 0.019881 | 4.38379125708e-169 | FAM117B | intronic | 11.93 |
| rs76639598 | 3 | 5800497 | rs76639598 | 0.023857 | 1e-200 | AC027119.1 | intergenic | 0.607 |
| rs77558416 | 3 | 31905786 | rs77558416 | 0.021869 | 5.59907198229e-120 | OSBPL10 | intronic | 11.15 |
| rs4676489 | 3 | 39349781 | rs4676489 | 0.018887 | 6.7467007693e-17 | RP11-331G2.6 | intergenic | 2.037 |
| rs376612740 | 3 | 112382391 | rs376612740 | 0.078529 | 1.92247691954e-189 | CCDC80 | intergenic | 1.329 |
| rs10935335 | 3 | 139294272 | rs10935335 | 0.038767 | 1e-200 | RP11-319G6.1:NMNAT3 | ncRNA_intronic | 1.591 |
| rs76083848 | 3 | 144933634 | rs76083848 | 0.025845 | 1e-200 | RP11-622L21.1 | intergenic | 1.469 |
| rs114775298 | 3 | 168503010 | rs114775298 | 0.026839 | 3.78736067236e-21 | EGFEM1P | ncRNA_intronic | 0.522 |
| rs62289859 | 3 | 184884679 | rs62289859 | 0.075547 | 1e-200 | EHHADH-AS1 | ncRNA_intronic | 0.186 |
| rs139543189 | 3 | 197196403 | rs139543189 | 0.020875 | 1.2070615681e-93 | AC128709.2 | intergenic | 2.997 |
| rs75807625 | 4 | 2723964 | rs75807625 | 0.023857 | 3.99681138515e-200 | FAM193A | intronic | 2.201 |
| rs61756977 | 4 | 47802162 | rs61756977 | 0.026839 | 1.36684487231e-17 | CORIN | intronic | 1.855 |
| rs150957872 | 4 | 72158409 | rs150957872 | 0.012923 | 1.31864134141e-29 | SLC4A4 | intronic | 1.554 |
| rs76870542 | 4 | 90612588 | rs76870542 | 0.026839 | 3.09027212201e-60 | RP11-115D19.1 | ncRNA_intronic | 0.703 |
| rs112201606 | 4 | 92612240 | rs112201606 | 0.025845 | 1e-200 | CCSER1 | intergenic | 1.173 |
| rs74332078 | 4 | 103766720 | rs74332078 | 0.036779 | 1e-200 | UBE2D3 | intronic | 0.456 |
| rs13137555 | 4 | 111150690 | rs13137555 | 0.018887 | 6.72487720798e-27 | HSBP1P2 | intergenic | 4.515 |
| rs74701742 | 4 | 121509613 | rs74701742 | 0.037773 | 1e-200 | RP11-501E14.1 | intergenic | 1.662 |
| rs115888840 | 4 | 173135565 | rs115888840 | 0.013917 | 1.2717934262e-10 | GALNTL6 | intronic | 1.742 |
| rs74534063 | 4 | 182949274 | rs74534063 | 0.076541 | 7.42428915757e-200 | AC108142.1 | ncRNA_intronic | 1.355 |
| rs72730669 | 5 | 37977668 | rs72730669 | 0.076541 | 1e-200 | CTD-2130F23.1 | intergenic | 0.488 |
| rs2337414 | 5 | 45568669 | rs2337414 | 0.077535 | 1e-200 | HCN1 | intronic | 5.193 |
| rs16878633 | 5 | 60504602 | rs16878633 | 0.025845 | 8.79428980684e-10 | CTC-436P18.1:CTC-436P18.3 | ncRNA_intronic | 7.812 |
| rs76958242 | 5 | 75041363 | rs76958242 | 0.076541 | 1e-200 | SLC25A5P9 | intergenic | 1.154 |
| rs16902844 | 5 | 87143483 | rs16902844 | 0.038767 | 1e-200 | CTD-2316B1.2 | intergenic | 8.484 |
| rs112112085 | 5 | 104085856 | rs112112085 | 0.026839 | 5.28129298344e-20 | RP11-6N13.1 | ncRNA_intronic | 4.159 |
| rs10067453 | 5 | 111933447 | rs10067453 | 0.026839 | 2.54635854392e-30 | RP11-159K7.2 | intergenic | 6.227 |
| rs77716125 | 5 | 141953310 | rs77716125 | 0.023857 | 1.72199033244e-133 | AC005592.2 | ncRNA_intronic | 3.537 |
| rs114875219 | 5 | 147460045 | rs114875219 | 0.023857 | 1.66806014992e-41 | SPINK5 | intronic | 0.257 |
| rs116570776 | 5 | 156835085 | rs116570776 | 0.015905 | 1.05741735402e-128 | CTB-109A12.1:ADAM19 | ncRNA_intronic | 2.412 |
| rs11743318 | 5 | 177605805 | rs11743318 | 0.077535 | 1e-200 | GMCL1P1 | intergenic | 6.029 |
| rs17078961 | 5 | 178457161 | rs17078961 | 0.019881 | 6.29161942451e-194 | ZNF879 | intronic | 6.147 |
| rs74781311 | 6 | 6839193 | rs74781311 | 0.026839 | 1.36831930837e-17 | BTF3P7 | intergenic | 4.293 |
| rs9392879 | 6 | 7345283 | rs9392879 | 0.035785 | 1.11428620997e-71 | SSR1:CAGE1 | intronic | 1 |
| rs17398435 | 6 | 7604106 | rs17398435 | 0.075547 | 1e-200 | SNRNP48 | intronic | 8.479 |
| rs62389223 | 6 | 12819405 | rs62389223 | 0.079523 | 1.13383050642e-189 | PHACTR1 | intronic | 5.126 |
| rs71554596 | 6 | 17010141 | rs71554596 | 0.076541 | 1e-200 | STMND1 | intergenic | 0.35 |
| rs7452568 | 6 | 23593581 | rs7452568 | 0.072565 | 1e-200 | RP4-810F7.1 | intergenic | 0.431 |
| rs7774931 | 6 | 54159874 | rs7774931 | 0.068589 | 1e-200 | TINAG | intergenic | 7.587 |
| rs141908879 | 6 | 66025248 | rs141908879 | 0.023857 | 1.34643847252e-109 | EYS | intronic | 1.637 |
| rs143468892 | 6 | 100089595 | rs143468892 | 0.014911 | 1e-200 | Y_RNA | downstream | 0.348 |
| rs113154201 | 6 | 107298354 | rs113154201 | 0.019881 | 6.14313357907e-12 | C6orf203 | intergenic | 1.451 |
| rs62439706 | 6 | 150179380 | rs62439706 | 0.035785 | 1e-200 | LRP11 | intronic | 5.73 |
| rs2128948 | 6 | 151661141 | rs2128948 | 0.036779 | 4.25147473015e-67 | AKAP12 | intronic | 1.579 |
| rs7761031 | 6 | 160695742 | rs7761031 | 0.039761 | 1e-200 | SLC22A2:RP1-276N6.2 | ncRNA_intronic | 4.341 |
| rs78108244 | 6 | 165385599 | rs78108244 | 0.020875 | 3.2584030226e-26 | RP11-300M24.1 | intergenic | 4.121 |
| rs35676495 | 7 | 1144767 | rs35676495 | 0.013917 | 1e-200 | C7orf50 | intronic | 3.236 |
| rs112772005 | 7 | 21528655 | rs112772005 | 0.025845 | 8.80856082266e-17 | SP4 | intronic | 12.7 |
| rs117481973 | 7 | 105841082 | rs117481973 | 0.021869 | 7.08294278445e-33 | RNU6-392P | intergenic | 6.131 |
| rs191056729 | 7 | 119752668 | rs191056729 | 0.025845 | 1.71432785651e-13 | RP4-742N3.1 | intergenic | 0.587 |
| rs117061478 | 7 | 123933855 | rs117061478 | 0.017893 | 1.08947965222e-09 | RP5-921G16.1 | ncRNA_intronic | 4.178 |
| rs56317486 | 7 | 124966934 | rs56317486 | 0.079523 | 3.3183659265e-189 | RP11-3B12.2 | ncRNA_intronic | 6.554 |
| rs17867320 | 7 | 126862398 | rs17867320 | 0.077535 | 1e-200 | GRM8:AC000099.1 | ncRNA_intronic | 2.525 |
| rs114504887 | 8 | 3751742 | rs114504887 | 0.016899 | 3.17470303684e-51 | CSMD1 | intronic | 0.903 |
| rs17717355 | 8 | 9198695 | rs17717355 | 0.070577 | 1e-200 | RP11-115J16.1 | ncRNA_intronic | 16.5 |
| rs78524955 | 8 | 18456065 | rs78524955 | 0.024851 | 1e-200 | PSD3 | intronic | 7.066 |
| rs36039502 | 8 | 21002414 | rs36039502 | 0.076541 | 1e-200 | AC021613.1 | intergenic | 8.77 |
| rs138859866 | 8 | 24098143 | rs138859866 | 0.013917 | 2.74872489019e-96 | ADAM28 | intergenic | 1.471 |
| rs72643032 | 8 | 37856779 | rs72643032 | 0.078529 | 1e-200 | KB-1836B5.3 | intergenic | 0.246 |
| rs192536583 | 8 | 65852335 | rs192536583 | 0.019881 | 1e-200 | RP11-89A16.1 | intergenic | 4.444 |
| rs28756805 | 8 | 95847357 | rs28756805 | 0.036779 | 1e-200 | INTS8 | intronic | 0.415 |
| rs117265439 | 8 | 109802217 | rs117265439 | 0.034791 | 4.04307786184e-25 | TMEM74 | intergenic | 0.069 |
| rs17729371 | 8 | 116708602 | rs17729371 | 0.069583 | 1e-200 | TRPS1 | intronic | 13.07 |
| rs35705938 | 8 | 120134308 | rs35705938 | 0.038767 | 1e-200 | COLEC10 | intergenic | 7.629 |
| rs1597280 | 8 | 134775266 | rs1597280 | 0.079523 | 1e-200 | CTD-2309H9.2 | intergenic | 1.374 |
| rs117719340 | 9 | 30887074 | rs117719340 | 0.023857 | 7.39345525355e-121 | FTLP4 | intergenic | 0.933 |
| rs149926044 | 9 | 87587550 | rs149926044 | 0.023857 | 1e-200 | NTRK2 | intronic | 0.18 |
| rs72747427 | 9 | 96413594 | rs72747427 | 0.075547 | 1e-200 | PHF2 | intronic | 4.936 |
| rs11787891 | 9 | 101677829 | rs11787891 | 0.076541 | 1e-200 | RP11-92C4.4 | intergenic | 1.038 |
| rs2026362 | 9 | 104240754 | rs2026362 | 0.078529 | 1e-200 | RP11-490D19.6:TMEM246 | ncRNA_intronic | 0.726 |
| rs62574705 | 9 | 121340169 | rs62574705 | 0.071571 | 1e-200 | RP11-349E4.1 | intergenic | 1.342 |
| rs68184458 | 9 | 129289638 | rs68184458 | 0.075547 | 1e-200 | RP11-205K6.2 | upstream | 0.831 |
| rs2277186 | 9 | 133543980 | rs2277186 | 0.076541 | 1e-200 | PRDM12 | intronic | 6.93 |
| rs192129994 | 9 | 135956068 | rs192129994 | 0.024851 | 2.41365146315e-09 | CELP | intergenic | 1.587 |
| rs147278971 | 9 | 136174495 | rs147278971 | 0.034791 | 6.40445114573e-61 | Y_RNA | intergenic | 2.118 |
| rs12779592 | 10 | 2378293 | rs12779592 | 0.014911 | 2.16856465607e-11 | LINC00701 | intergenic | 4.691 |
| rs9787428 | 10 | 22848007 | rs9787428 | 0.075547 | 1e-200 | PIP4K2A | intronic | 0.488 |
| rs150489002 | 10 | 26095093 | rs150489002 | 0.023857 | 1.21966937525e-151 | RNA5SP306 | intergenic | 1.852 |
| rs4745982 | 10 | 71089843 | rs4745982 | 0.075547 | 1e-200 | HK1 | intronic | 4.295 |
| rs111532642 | 10 | 81089851 | rs111532642 | 0.026839 | 9.13613571236e-19 | ZMIZ1 | intergenic | 2.013 |
| rs61854881 | 10 | 90878904 | rs61854881 | 0.018887 | 9.59789530733e-110 | RP11-341B24.3 | intergenic | 4.763 |
| rs41286940 | 10 | 93609210 | rs41286940 | 0.079523 | 1.86631649615e-188 | TNKS2 | intronic | 5.821 |
| rs56187102 | 10 | 95375783 | rs56187102 | 0.026839 | 3.78935806163e-21 | PDE6C | intronic | 0.202 |
| rs61736835 | 10 | 95641139 | rs61736835 | 0.036779 | 1e-200 | RAB11AP1 | ncRNA_exonic | 0.033 |
| rs183518579 | 10 | 100862091 | rs183518579 | 0.026839 | 3.30617626311e-32 | HPSE2 | intronic | 4.712 |
| rs78491197 | 10 | 117937634 | rs78491197 | 0.017893 | 7.7004524743e-64 | GFRA1 | intronic | 2.952 |
| rs117973923 | 11 | 25311104 | rs117973923 | 0.024851 | 1.77985345929e-85 | RP11-54J7.2 | intergenic | 5.78 |
| rs11037543 | 11 | 43680111 | rs11037543 | 0.073559 | 1e-200 | HSD17B12 | intergenic | 4.928 |
| rs79999706 | 11 | 96748968 | rs79999706 | 0.078529 | 1e-200 | MED28P5 | intergenic | 4.003 |
| rs3016356 | 11 | 116477772 | rs3016356 | 0.069583 | 1e-200 | AP000770.1 | intergenic | 4.475 |
| rs75542613 | 11 | 116679155 | rs75542613;rs1135663 | 0.071571 | 1e-200 | AP006216.5 | intergenic | 4.182 |
| rs59781045 | 11 | 117095283 | rs59781045 | 0.071571 | 1e-200 | PCSK7 | intronic | 1.757 |
| rs28990986 | 11 | 118963291 | rs28990986 | 0.074553 | 1e-200 | HMBS | intronic | 6.29 |
| rs367998292 | 11 | 121727848 | rs367998292;rs12280388 | 0.077535 | 1e-200 | RNU6-256P | intergenic | 8.516 |
| rs145765147 | 12 | 7891492 | rs145765147 | 0.035785 | 2.20727836441e-23 | CLEC4C | intronic | 1.894 |
| rs56058261 | 12 | 12544111 | rs56058261 | 0.021869 | 5.9006360688e-58 | LOH12CR1 | intronic | 0.378 |
| rs74588265 | 12 | 21020988 | rs74588265 | 0.021869 | 1.10472737793e-95 | SLCO1B3:LST3:SLCO1B7 | intronic | 8.985 |
| rs73088639 | 12 | 51312457 | rs73088639 | 0.014911 | 2.08044153328e-25 | METTL7A | intergenic | 9.111 |
| rs61924767 | 12 | 65504149 | rs61924767 | 0.016899 | 1.94279510159e-194 | WIF1 | intronic | 0.396 |
| rs144814086 | 12 | 76689666 | rs144814086 | 0.015905 | 2.96192878683e-18 | RP11-54A9.1 | ncRNA_intronic | 4.267 |
| rs117178443 | 12 | 77122002 | rs117178443 | 0.071571 | 1e-200 | RPL7P43 | intergenic | 1.279 |
| rs79168361 | 12 | 97383403 | rs79168361 | 0.010934 | 2.58853851795e-13 | RP11-541G9.1 | ncRNA_intronic | 3.705 |
| rs146929492 | 12 | 100484751 | rs146929492 | 0.013917 | 1.74697235844e-18 | UHRF1BP1L | intronic | 0.021 |
| rs2293432 | 12 | 112601808 | rs2293432 | 0.077535 | 1e-200 | HECTD4 | intronic | 1.914 |
| rs142268019 | 13 | 70784181 | rs142268019 | 0.026839 | 9.24432208015e-26 | ATXN8OS | intergenic | 0.418 |
| rs7318349 | 13 | 72973843 | rs7318349 | 0.079523 | 1.27096636677e-197 | SNORD37 | intergenic | 0.188 |
| rs17110331 | 14 | 26194481 | rs17110331 | 0.077535 | 1e-200 | SNORD37 | intergenic | 0.68 |
| rs140258097 | 14 | 82250557 | rs140258097 | 0.036779 | 2.01145332194e-22 | RP11-666E17.1 | ncRNA_intronic | 1.828 |
| rs78430956 | 14 | 92210640 | rs78430956 | 0.026839 | 2.52788835235e-30 | CATSPERB | intronic | 0.197 |
| rs79881262 | 14 | 98745164 | rs79881262 | 0.037773 | 1e-200 | AL163760.1 | intergenic | 1.011 |
| rs145691643 | 15 | 48812477 | rs145691643 | 0.023857 | 1e-200 | FBN1 | intronic | 0.589 |
| rs138380023 | 15 | 58097304 | rs138380023 | 0.026839 | 5.44498057554e-29 | POLR2M | intergenic | 0.73 |
| rs4646583 | 15 | 58306551 | rs4646583 | 0.075547 | 1e-200 | ALDH1A2 | intronic | 3.176 |
| rs77027049 | 15 | 58716377 | rs77027049 | 0.072565 | 1e-200 | ALDH1A2:LIPC | intronic | 2.53 |
| rs4776293 | 15 | 66530274 | rs4776293 | 0.021869 | 1e-200 | MEGF11 | intronic | 13.74 |
| rs8048774 | 16 | 1438973 | rs8048774 | 0.023857 | 1.60628416635e-128 | UNKL | intronic | 0.346 |
| rs145940844 | 16 | 4845575 | rs145940844 | 0.018887 | 1e-200 | SMIM22:RP11-127I20.5 | ncRNA_exonic | 0.047 |
| rs78763534 | 16 | 6089969 | rs78763534 | 0.078529 | 1e-200 | RP11-420N3.2:RBFOX1 | ncRNA_intronic | 8.146 |
| rs111837854 | 16 | 7041673 | rs111837854 | 0.072565 | 1e-200 | RBFOX1 | intronic | 1.019 |
| rs79322314 | 16 | 74883956 | rs79322314 | 0.013917 | 1e-200 | RP11-787D11.1 | intergenic | 2.185 |
| rs28379208 | 16 | 84011209 | rs28379208 | 0.016899 | 4.46153008072e-92 | NECAB2 | intronic | 1.744 |
| rs9940232 | 16 | 87584974 | rs9940232 | 0.017893 | 3.36336874997e-103 | RP11-482M8.1 | intergenic | 2.469 |
| rs77680021 | 17 | 7124506 | rs77680021 | 0.075547 | 3.90173837435e-200 | ACADVL | intronic | 0.376 |
| rs76868109 | 17 | 41776943 | rs76868109 | 0.040756 | 1e-200 | CTC-501O10.1 | ncRNA_intronic | 12.5 |
| rs113741838 | 17 | 49029897 | rs113741838 | 0.073559 | 1e-200 | RP11-700H6.2 | intergenic | 3.033 |
| rs17700212 | 17 | 65107857 | rs17700212 | 0.022863 | 4.60258981089e-199 | HELZ | intronic | 0.451 |
| rs77293315 | 18 | 24702245 | rs77293315 | 0.016899 | 3.85110817132e-48 | AQP4-AS1:CHST9 | ncRNA_intronic | 5.942 |
| rs146815093 | 18 | 26654035 | rs146815093 | 0.011928 | 1.29376965258e-09 | AC090349.1 | intergenic | 0.917 |
| rs17068330 | 18 | 59134473 | rs17068330 | 0.024851 | 1e-200 | CDH20 | intronic | 6.24 |
| rs75152146 | 18 | 60437638 | rs75152146 | 0.018887 | 1.2700626339e-18 | PHLPP1 | intronic | 0.102 |
| rs150172415 | 18 | 62125113 | rs150172415 | 0.036779 | 1.88680402274e-29 | RP11-146N18.1 | intergenic | 0.781 |
| rs117831057 | 19 | 10707853 | rs117831057 | 0.071571 | 1e-200 | CTC-539A10.7 | downstream | 1.752 |
| rs56322906 | 19 | 11346155 | rs56322906;rs3745683 | 0.042744 | 1e-200 | DOCK6 | intronic | 6.462 |
| rs16995852 | 19 | 19112765 | rs16995852 | 0.039761 | 1e-200 | SUGP2 | intronic | 0.344 |
| rs117414940 | 19 | 19855549 | rs117414940 | 0.033797 | 4.55492914682e-37 | CTC-559E9.9 | intergenic | 0.335 |
| rs62116889 | 19 | 45022560 | rs62116889 | 0.087475 | 2.74750902394e-20 | CEACAM20 | ncRNA_intronic | 0.917 |
| rs112450640 | 19 | 45296364 | rs112450640 | 0.015905 | 1.78050830199e-14 | CBLC | intronic | 0.417 |
| rs117803469 | 20 | 1600405 | rs117803469 | 0.035785 | 1.12284511303e-39 | RP4-576H24.4:SIRPB1 | intronic | 5.894 |
| rs56219025 | 20 | 25239801 | rs56219025 | 0.073559 | 1e-200 | PYGB | intronic | 3.694 |
| rs80304109 | 20 | 32739552 | rs80304109 | 0.035785 | 4.09711849856e-27 | RPS2P1 | intergenic | 2.288 |
| rs79906004 | 20 | 35854324 | rs79906004 | 0.026839 | 2.3287504327e-18 | RPN2 | intronic | 0.086 |
| rs117162746 | 20 | 38699715 | rs117162746 | 0.075547 | 1e-200 | RP11-101E14.3 | intergenic | 5.148 |
| rs6130478 | 20 | 42533655 | rs6130478 | 0.024851 | 1.13953689156e-08 | RP5-1030M6.3 | intergenic | 1.057 |
| rs6121704 | 20 | 60153026 | rs6121704 | 0.019881 | 1e-200 | CDH4 | intronic | 4.426 |
| rs75978011 | 21 | 23007063 | rs75978011 | 0.037773 | 1e-200 | AF241725.1 | upstream | 2.092 |
| rs73229346 | 21 | 23253308 | rs73229346 | 0.072565 | 1e-200 | AP000472.3 | intergenic | 3.454 |
| rs73905204 | 21 | 36609451 | rs73905204 | 0.024851 | 1e-200 | RUNX1 | intronic | 0.04 |
| rs12053781 | 22 | 26581926 | rs12053781 | 0.036779 | 1e-200 | SEZ6L | intronic | 4.356 |
| rs116923341 | 22 | 35259690 | rs116923341 | 0.019881 | 4.8292505025e-91 | RP1-272J12.1 | intergenic | 4.712 |
| rs73179581 | 22 | 49290633 | rs73179581 | 0.071571 | 1e-200 | WI2-81516E3.1 | ncRNA_exonic | 0.741 |

**Supplementary Table 9.** Results of finemap analysis

| **Locus** | **SNP** | **CHR** | **POS** | **MAF** | **P** | **tstat** | **leadSNP** | **SUSIE.CS** | **SUSIE.PP** | **FINEMAP.CS** | **FINEMAP.PP** | **FINEMAP.k** | **mean.PP** |
| --- | --- | --- | --- | --- | --- | --- | --- | --- | --- | --- | --- | --- | --- |
| ALDH1A2_LIPC | rs77027049 | 15 | 58716377 | 0.07 | 1e-200 | 32.22673 | TRUE | 1 | 1 | 1 | 1 | 5 | 1 |
| AP000472.3 | rs75978011 | 21 | 23007063 | 0.04 | 1e-200 | 42.10839 | TRUE | 1 | 1 | 0 | 1 | 2 | 1 |
| CBLC | rs2965148 | 19 | 45190773 | 0.46 | 4.99e-06 | -4.56505 | FALSE | 1 | 1 | 0 | 1 | 5 | 1 |
| FAM117B | rs116126337 | 2 | 203525422 | 0.02 | 4.38e-169 | -27.7168 | TRUE | 1 | 1 | 0 | 1 | 5 | 1 |
| FAM117B | rs138672613 | 2 | 203318618 | 0.03 | 0.68093 | 0.411194 | FALSE | 2 | 1 | 0 | 1 | 5 | 1 |
| FBN1 | rs145691643 | 15 | 48812477 | 0.02 | 1e-200 | -35.5725 | TRUE | 1 | 1 | 1 | 1 | 5 | 1 |
| FBN1 | rs62011404 | 15 | 48830657 | 0.03 | 0.42986 | -0.78943 | FALSE | 4 | 1 | 1 | 1 | 5 | 1 |
| FOXD2 | rs190672573 | 1 | 47940650 | 0.01 | 0.987357 | 0.015846 | FALSE | 2 | 1 | 0 | 1 | 5 | 1 |
| FOXD2 | rs41290150 | 1 | 47901586 | 0.01 | 1e-200 | 66.97881 | TRUE | 1 | 1 | 0 | 1 | 5 | 1 |
| PIP4K2A | rs7900881 | 10 | 22832901 | 0.06 | 0.471782 | 0.719583 | FALSE | 3 | 1 | 1 | 1 | 5 | 1 |
| PIP4K2A | rs9787428 | 10 | 22848007 | 0.08 | 1e-200 | -33.5639 | TRUE | 1 | 1 | 1 | 1 | 5 | 1 |
| PRDM12 | rs2277186 | 9 | 133543980 | 0.08 | 1e-200 | 32.3898 | TRUE | 1 | 1 | 1 | 1 | 5 | 1 |
| TMEM74 | rs116936361 | 8 | 109681920 | 0.03 | 0.419318 | 0.807604 | FALSE | 2 | 1 | 0 | 1 | 5 | 1 |
| TMEM74 | rs117265439 | 8 | 109802217 | 0.03 | 4.04e-25 | -10.3533 | TRUE | 3 | 1 | 0 | 1 | 5 | 1 |
| WI2-81516E3.1 | rs73179581 | 22 | 49290633 | 0.07 | 1e-200 | 40.80327 | TRUE | 1 | 1 | 0 | 1 | 5 | 1 |

**Supplementary Table 10.** Significant genes identified by FUSION

| **PANEL** | **ID** | **CHR** | **BEST.GWAS.ID** | **BEST.GWAS.Z** | **EQTL.ID** | **EQTL.R2** | **EQTL.Z** | **EQTL.GWAS.Z** | **TWAS.Z** | **TWAS.P** |
| --- | --- | --- | --- | --- | --- | --- | --- | --- | --- | --- |
| sCCA2 | ENSG00000268030.1 | 19 | rs16995852 | 53.18 | rs2013813 | 0.00898 | -4.29 | -24.9 | 31.62652 | 1.6e-219 |
| sCCA2 | ENSG00000162702.7 | 1 | rs12070749 | 38.36 | rs12070749 | 0.0508 | 5.09 | 38.35721 | 28.36505 | 5.46e-177 |
| sCCA3 | ENSG00000268030.1 | 19 | rs16995852 | 53.18 | rs2013813 | 0.00536 | -3.95 | -24.9 | 26 | 9.2e-149 |
| sCCA2 | ENSG00000167487.11 | 19 | rs16995852 | 53.18 | rs12971493 | 0.0571 | -4.63 | -25.8 | 25.77686 | 1.61e-146 |
| sCCA3 | ENSG00000214212.8 | 19 | rs3745683 | 32.83 | rs2278426 | 0.0832 | 5.22 | 25.7 | 25.7 | 3.87e-145 |
| sCCA3 | ENSG00000105700.10 | 19 | rs16995852 | 53.18 | rs1859948 | 0.0293 | -4.83 | 1.07 | -10.6 | 4e-26 |
| sCCA3 | ENSG00000006015.17 | 19 | rs16995852 | 53.18 | rs4808823 | 0.0037 | 4.35 | -0.61 | 10.5 | 5.33e-26 |
| sCCA3 | ENSG00000184162.14 | 19 | rs16995852 | 53.18 | rs10410399 | 0.00281 | 3.99 | -23.8 | -9.25 | 2.21e-20 |
| sCCA1 | ENSG00000268030.1 | 19 | rs16995852 | 53.18 | rs2057649 | 0.0247 | 4.6 | -0.747 | 8.943653 | 3.77e-19 |
| sCCA2 | ENSG00000105671.11 | 19 | rs16995852 | 53.18 | rs6511021 | 0.019 | -4.48 | -0.156 | -8.53752 | 1.37e-17 |
| sCCA3 | ENSG00000064607.16 | 19 | rs16995852 | 53.18 | rs10417318 | 0.0614 | -4.93 | 1.16 | 7.76 | 8.48e-15 |
| sCCA1 | ENSG00000105700.10 | 19 | rs16995852 | 53.18 | rs2314664 | 0.139 | -6.79 | -0.683 | 7.506091 | 6.09e-14 |
| sCCA1 | ENSG00000064607.16 | 19 | rs16995852 | 53.18 | rs4808171 | 0.0262 | -5 | -0.614 | -6.72754 | 1.73e-11 |
| sCCA2 | ENSG00000124784.8 | 6 | rs17398435 | -37.17 | rs3818425 | 0.130058 | -7.65 | 0.31551 | -6.42765 | 1.3e-10 |
| sCCA2 | ENSG00000105700.10 | 19 | rs16995852 | 53.18 | rs1859948 | 0.00101 | -4.18 | 1.07 | -6.40994 | 1.46e-10 |
| sCCA3 | ENSG00000205517.12 | 19 | rs3745683 | 32.83 | rs34095 | 0.0528 | -4.17 | -7.59 | 6.17 | 6.65e-10 |
| sCCA3 | ENSG00000064545.14 | 19 | rs16995852 | 53.18 | rs10417318 | 0.159 | 7.29 | 1.16 | 5.97 | 2.37e-09 |
| sCCA1 | ENSG00000105671.11 | 19 | rs16995852 | 53.18 | rs2269806 | 0.0648 | 5.42 | 0.846 | 5.667238 | 1.45e-08 |
| sCCA2 | ENSG00000124783.12 | 6 | rs17398435 | -37.17 | rs10901004 | 0.008429 | 4.45 | -0.54202 | 4.98602 | 4.16e-08 |

**Supplementary Table 11.** Detailed mutation information of ZNF281 and RIOK1

| **Gene** | **Position** | **Replacement** | **Original** | **Type** | **SNP** |
| --- | --- | --- | --- | --- | --- |
| ZNF281 | 280 | C | A | Missense variant | rs2102483805 |
| RIOK1 | 384 | G | T | Missense variant | rs200129596 |

**Supplementary Table 12.** Mendelian randomization results for 50055 traits in the IEU database (FDR correction)

| **Method** | **outcome** | **exposure** | **nsnp** | **b** | **se** | **pval** | **Q** | **Q_df** | **Q_pval** | **FDR** |
| --- | --- | --- | --- | --- | --- | --- | --- | --- | --- | --- |
| Inverse variance weighted | PSYCH_factor | ebi-a-GCST90029008 | 4778 | 0.011149 | 0.001191 | 7.61E-21 | 3767.975 | 4777 | 1 | 8.34E-16 |
| Inverse variance weighted | PSYCH_factor | ebi-a-GCST90029025 | 1659 | 0.027316 | 0.002995 | 7.41E-20 | 902.2851 | 1658 | 1 | 4.06E-15 |
| Inverse variance weighted | PSYCH_factor | ukb-b-10787 | 3461 | 0.015078 | 0.00168 | 2.91E-19 | 3023.245 | 3460 | 0.999999979 | 1.06E-14 |
| Inverse variance weighted | PSYCH_factor | ukb-b-14540 | 1466 | 0.027222 | 0.003092 | 1.32E-18 | 835.6359 | 1465 | 1 | 2.19E-14 |
| Inverse variance weighted | PSYCH_factor | ukb-b-13354 | 1477 | 0.02724 | 0.00308 | 9.32E-19 | 831.4396 | 1476 | 1 | 2.19E-14 |
| Inverse variance weighted | PSYCH_factor | ukb-b-17409 | 1511 | 0.026791 | 0.003045 | 1.40E-18 | 855.615 | 1510 | 1 | 2.19E-14 |
| Inverse variance weighted | PSYCH_factor | ukb-b-9685 | 1494 | 0.02712 | 0.003079 | 1.26E-18 | 852.4651 | 1493 | 1 | 2.19E-14 |
| Inverse variance weighted | PSYCH_factor | ukb-b-16446 | 1405 | 0.026153 | 0.003047 | 9.29E-18 | 796.8822 | 1404 | 1 | 1.27E-13 |
| Inverse variance weighted | PSYCH_factor | ukb-b-19925 | 1227 | 0.027966 | 0.003432 | 3.66E-16 | 697.0624 | 1226 | 1 | 4.37E-12 |
| Inverse variance weighted | PSYCH_factor | ebi-a-GCST90025949 | 3639 | 0.010827 | 0.00133 | 3.99E-16 | 2166.482 | 3638 | 1 | 4.37E-12 |
| Inverse variance weighted | PSYCH_factor | ukb-b-9093 | 1198 | 0.027594 | 0.003452 | 1.32E-15 | 668.8622 | 1197 | 1 | 1.32E-11 |
| Inverse variance weighted | PSYCH_factor | ebi-a-GCST90018959 | 2702 | 0.013691 | 0.001798 | 2.61E-14 | 1601.149 | 2701 | 1 | 2.04E-10 |
| Inverse variance weighted | PSYCH_factor | ebi-a-GCST90092960 | 102 | 0.009762 | 0.001284 | 2.92E-14 | 96.8868 | 101 | 0.597289946 | 2.13E-10 |
| Inverse variance weighted | PSYCH_factor | ukb-b-19520 | 1210 | 0.026584 | 0.003508 | 3.48E-14 | 684.5243 | 1209 | 1 | 2.39E-10 |
| Inverse variance weighted | PSYCH_factor | met-d-Total_FC | 112 | 0.015371 | 0.002054 | 7.28E-14 | 114.7258 | 111 | 0.385161116 | 4.20E-10 |
| Inverse variance weighted | PSYCH_factor | ukb-b-16698 | 1195 | 0.026166 | 0.003512 | 9.32E-14 | 667.2541 | 1194 | 1 | 5.10E-10 |
| Inverse variance weighted | PSYCH_factor | ukb-b-16099 | 1204 | 0.024276 | 0.00333 | 3.09E-13 | 705.1673 | 1203 | 1 | 1.23E-09 |
| Inverse variance weighted | PSYCH_factor | ukb-b-17271 | 1222 | 0.024334 | 0.003331 | 2.76E-13 | 718.526 | 1221 | 1 | 1.23E-09 |
| Inverse variance weighted | PSYCH_factor | ebi-a-GCST90000025 | 2058 | 0.011725 | 0.001626 | 5.50E-13 | 1204.053 | 2057 | 1 | 1.83E-09 |
| Inverse variance weighted | PSYCH_factor | ukb-b-14310 | 1227 | 0.023854 | 0.003317 | 6.41E-13 | 708.7983 | 1226 | 1 | 1.95E-09 |
| Inverse variance weighted | PSYCH_factor | ebi-a-GCST90092838 | 92 | 0.011911 | 0.001662 | 7.62E-13 | 86.78826 | 91 | 0.605461984 | 2.09E-09 |
| Inverse variance weighted | PSYCH_factor | ebi-a-GCST90092985 | 105 | 0.014473 | 0.002022 | 8.12E-13 | 109.3283 | 104 | 0.34113175 | 2.12E-09 |
| Inverse variance weighted | PSYCH_factor | ukb-b-12828 | 1229 | 0.023544 | 0.00329 | 8.35E-13 | 720.3871 | 1228 | 1 | 2.13E-09 |
| Inverse variance weighted | PSYCH_factor | ukb-a-292 | 842 | 0.024215 | 0.003417 | 1.37E-12 | 472.4293 | 841 | 1 | 3.08E-09 |
| Inverse variance weighted | PSYCH_factor | ukb-a-389 | 1980 | 0.012491 | 0.001762 | 1.37E-12 | 1182.394 | 1979 | 1 | 3.08E-09 |
| Inverse variance weighted | PSYCH_factor | ukb-a-293 | 839 | 0.02434 | 0.003436 | 1.41E-12 | 460.7755 | 838 | 1 | 3.09E-09 |
| Inverse variance weighted | PSYCH_factor | met-d-non_HDL_C | 102 | 0.013672 | 0.00199 | 6.39E-12 | 124.256 | 101 | 0.058050027 | 1.17E-08 |
| Inverse variance weighted | PSYCH_factor | ukb-a-266 | 815 | 0.023785 | 0.003492 | 9.67E-12 | 442.6892 | 814 | 1 | 1.66E-08 |
| Inverse variance weighted | PSYCH_factor | ukb-a-267 | 794 | 0.023705 | 0.003526 | 1.77E-11 | 429.0307 | 793 | 1 | 2.86E-08 |
| Inverse variance weighted | PSYCH_factor | met-d-L_LDL_L | 99 | 0.012202 | 0.00182 | 2.01E-11 | 120.2176 | 98 | 0.063309785 | 3.19E-08 |
| Inverse variance weighted | PSYCH_factor | ukb-a-268 | 737 | 0.02369 | 0.003556 | 2.69E-11 | 395.1542 | 736 | 1 | 4.22E-08 |
| Inverse variance weighted | PSYCH_factor | met-d-L_LDL_PL | 93 | 0.012065 | 0.001814 | 2.89E-11 | 112.4308 | 92 | 0.072759936 | 4.46E-08 |
| Inverse variance weighted | PSYCH_factor | met-d-S_LDL_P | 107 | 0.012324 | 0.001891 | 7.11E-11 | 123.8397 | 106 | 0.113572434 | 1.03E-07 |
| Inverse variance weighted | PSYCH_factor | met-d-Clinical_LDL_C | 95 | 0.011034 | 0.001704 | 9.35E-11 | 116.0251 | 94 | 0.061364202 | 1.30E-07 |
| Inverse variance weighted | PSYCH_factor | ebi-a-GCST90092864 | 90 | 0.011443 | 0.00179 | 1.63E-10 | 110.3352 | 89 | 0.062297168 | 2.13E-07 |
| Inverse variance weighted | PSYCH_factor | ukb-b-11842 | 1076 | 0.017092 | 0.002678 | 1.76E-10 | 605.0489 | 1075 | 1 | 2.24E-07 |
| Inverse variance weighted | PSYCH_factor | ukb-b-12039 | 1057 | 0.016936 | 0.002688 | 2.98E-10 | 589.8671 | 1056 | 1 | 3.67E-07 |
| Inverse variance weighted | PSYCH_factor | ukb-a-277 | 659 | 0.023913 | 0.003798 | 3.05E-10 | 362.5791 | 658 | 1 | 3.71E-07 |
| Inverse variance weighted | PSYCH_factor | ukb-a-288 | 666 | 0.024717 | 0.003929 | 3.16E-10 | 355.4608 | 665 | 1 | 3.73E-07 |
| Inverse variance weighted | PSYCH_factor | ukb-a-284 | 674 | 0.024874 | 0.004006 | 5.31E-10 | 385.1693 | 673 | 1 | 6.00E-07 |
| Inverse variance weighted | PSYCH_factor | ukb-a-276 | 661 | 0.023273 | 0.003768 | 6.55E-10 | 360.9633 | 660 | 1 | 7.25E-07 |
| Inverse variance weighted | PSYCH_factor | ebi-a-GCST90092969 | 145 | 0.010383 | 0.001699 | 9.94E-10 | 135.7279 | 144 | 0.676383453 | 1.06E-06 |
| Inverse variance weighted | PSYCH_factor | ukb-b-16881 | 1942 | 0.012728 | 0.002085 | 1.03E-09 | 1183.911 | 1941 | 1 | 1.08E-06 |
| Inverse variance weighted | PSYCH_factor | ukb-a-289 | 659 | 0.023969 | 0.003956 | 1.37E-09 | 362.9478 | 658 | 1 | 1.38E-06 |
| Inverse variance weighted | PSYCH_factor | ukb-a-35 | 749 | 0.019236 | 0.003188 | 1.60E-09 | 428.2418 | 748 | 1 | 1.60E-06 |
| Inverse variance weighted | PSYCH_factor | ukb-a-280 | 640 | 0.023193 | 0.003849 | 1.69E-09 | 354.0784 | 639 | 1 | 1.64E-06 |
| Inverse variance weighted | PSYCH_factor | ukb-a-285 | 665 | 0.02393 | 0.004001 | 2.21E-09 | 360.6195 | 664 | 1 | 2.12E-06 |
| Inverse variance weighted | PSYCH_factor | ebi-a-GCST90002405 | 1069 | 0.010554 | 0.001787 | 3.47E-09 | 647.7429 | 1068 | 1 | 3.20E-06 |
| Inverse variance weighted | PSYCH_factor | ebi-a-GCST90018949 | 860 | 0.017608 | 0.002993 | 4.04E-09 | 481.1496 | 859 | 1 | 3.63E-06 |
| Inverse variance weighted | PSYCH_factor | ukb-a-281 | 641 | 0.022696 | 0.003872 | 4.59E-09 | 359.3333 | 640 | 1 | 4.09E-06 |
| Inverse variance weighted | PSYCH_factor | ebi-a-GCST90000026 | 643 | 0.0121 | 0.002094 | 7.54E-09 | 367.685 | 642 | 1 | 6.22E-06 |
| Inverse variance weighted | PSYCH_factor | ebi-a-GCST90092994 | 99 | 0.018592 | 0.003219 | 7.68E-09 | 78.69257 | 98 | 0.924111583 | 6.28E-06 |
| Inverse variance weighted | PSYCH_factor | ebi-a-GCST90092919 | 150 | 0.010351 | 0.001833 | 1.64E-08 | 118.7878 | 149 | 0.967512627 | 1.30E-05 |
| Inverse variance weighted | PSYCH_factor | ebi-a-GCST90002406 | 1077 | 0.009528 | 0.001725 | 3.33E-08 | 648.6278 | 1076 | 1 | 2.48E-05 |
| Inverse variance weighted | PSYCH_factor | ebi-a-GCST90028997 | 1376 | -0.00882 | 0.001623 | 5.61E-08 | 827.5641 | 1375 | 1 | 4.07E-05 |
| Inverse variance weighted | PSYCH_factor | ebi-a-GCST90000027 | 983 | 0.010023 | 0.001874 | 8.92E-08 | 567.4489 | 982 | 1 | 6.39E-05 |
| Inverse variance weighted | PSYCH_factor | ukb-a-195 | 1148 | 0.012099 | 0.002274 | 1.04E-07 | 710.147 | 1147 | 1 | 7.33E-05 |
| Inverse variance weighted | PSYCH_factor | ebi-a-GCST90002340 | 1591 | -0.00777 | 0.001471 | 1.31E-07 | 968.5938 | 1590 | 1 | 8.99E-05 |
| Inverse variance weighted | PSYCH_factor | ukb-d-30250_irnt | 594 | 0.011748 | 0.00225 | 1.78E-07 | 370.3404 | 593 | 1 | 1.19E-04 |
| Inverse variance weighted | PSYCH_factor | ieu-b-31 | 1599 | -0.00769 | 0.001486 | 2.32E-07 | 982.5836 | 1598 | 1 | 1.48E-04 |
| Inverse variance weighted | PSYCH_factor | ebi-a-GCST90029000 | 1512 | 0.007728 | 0.001542 | 5.37E-07 | 971.879 | 1511 | 1 | 3.22E-04 |
| Inverse variance weighted | PSYCH_factor | ebi-a-GCST90025972 | 1033 | 0.009546 | 0.001914 | 6.13E-07 | 633.209 | 1032 | 1 | 3.65E-04 |
| Inverse variance weighted | PSYCH_factor | ebi-a-GCST90092971 | 138 | 0.00976 | 0.001968 | 7.09E-07 | 132.364 | 137 | 0.595955731 | 4.16E-04 |
| Inverse variance weighted | PSYCH_factor | ebi-a-GCST90028998 | 1382 | -0.00808 | 0.001632 | 7.34E-07 | 837.2323 | 1381 | 1 | 4.26E-04 |
| Inverse variance weighted | PSYCH_factor | met-d-HDL_FC | 183 | 0.012274 | 0.002481 | 7.52E-07 | 137.3437 | 182 | 0.994288618 | 4.34E-04 |
| Inverse variance weighted | PSYCH_factor | met-d-S_VLDL_L | 142 | 0.014986 | 0.003036 | 8.01E-07 | 129.4349 | 141 | 0.748088326 | 4.57E-04 |
| Inverse variance weighted | PSYCH_factor | ebi-a-GCST90019470 | 42 | -0.0056 | 0.001139 | 8.92E-07 | 38.74063 | 41 | 0.571500128 | 4.96E-04 |
| Inverse variance weighted | PSYCH_factor | met-d-Unsaturation | 109 | 0.015385 | 0.003132 | 9.02E-07 | 101.2804 | 108 | 0.663279116 | 4.99E-04 |
| Inverse variance weighted | PSYCH_factor | ebi-a-GCST90013983 | 1101 | -0.00833 | 0.001726 | 1.39E-06 | 655.3291 | 1100 | 1 | 7.40E-04 |
| Inverse variance weighted | PSYCH_factor | met-d-S_VLDL_P | 138 | 0.01441 | 0.002991 | 1.45E-06 | 123.2336 | 137 | 0.794073073 | 7.63E-04 |
| Inverse variance weighted | PSYCH_factor | ebi-a-GCST90092974 | 135 | 0.014829 | 0.003078 | 1.45E-06 | 126.9172 | 134 | 0.65527362 | 7.63E-04 |
| Inverse variance weighted | PSYCH_factor | met-d-S_VLDL_CE_pct | 139 | 0.009407 | 0.00198 | 2.01E-06 | 143.2593 | 138 | 0.362173328 | 1.02E-03 |
| Inverse variance weighted | PSYCH_factor | ebi-a-GCST002222 | 145 | 0.011137 | 0.002418 | 4.11E-06 | 156.15 | 144 | 0.230933455 | 1.98E-03 |
| Inverse variance weighted | PSYCH_factor | ebi-a-GCST002221 | 166 | 0.010967 | 0.00243 | 6.37E-06 | 162.5957 | 165 | 0.538338487 | 2.98E-03 |
| Inverse variance weighted | PSYCH_factor | ebi-a-GCST90019428 | 49 | -0.00477 | 0.001061 | 6.83E-06 | 49.52681 | 48 | 0.412103462 | 3.19E-03 |
| Inverse variance weighted | PSYCH_factor | ebi-a-GCST90093001 | 132 | 0.014204 | 0.00316 | 6.97E-06 | 122.6727 | 131 | 0.685934589 | 3.24E-03 |
| Inverse variance weighted | PSYCH_factor | ukb-b-8338 | 753 | 0.012864 | 0.002865 | 7.13E-06 | 403.3395 | 752 | 1 | 3.28E-03 |
| Inverse variance weighted | PSYCH_factor | ieu-a-301 | 165 | 0.010906 | 0.002431 | 7.26E-06 | 163.1229 | 164 | 0.504658168 | 3.33E-03 |
| Inverse variance weighted | PSYCH_factor | ukb-a-249 | 592 | 0.013743 | 0.003077 | 7.98E-06 | 318.7072 | 591 | 1 | 3.63E-03 |
| Inverse variance weighted | PSYCH_factor | met-d-VLDL_PL | 134 | 0.014109 | 0.00317 | 8.55E-06 | 127.5891 | 133 | 0.616133651 | 3.83E-03 |
| Inverse variance weighted | PSYCH_factor | ebi-a-GCST90092975 | 137 | 0.013186 | 0.002962 | 8.52E-06 | 126.7152 | 136 | 0.703899027 | 3.83E-03 |
| Inverse variance weighted | PSYCH_factor | ukb-b-7212 | 755 | 0.015916 | 0.003595 | 9.54E-06 | 408.6588 | 754 | 1 | 4.23E-03 |
| Inverse variance weighted | PSYCH_factor | met-c-845 | 24 | 0.015544 | 0.003522 | 1.02E-05 | 21.45296 | 23 | 0.553446462 | 4.48E-03 |
| Inverse variance weighted | PSYCH_factor | ukb-b-18096 | 733 | 0.015877 | 0.003612 | 1.11E-05 | 390.8001 | 732 | 1 | 4.83E-03 |
| Inverse variance weighted | PSYCH_factor | ukb-d-30240_irnt | 613 | 0.009368 | 0.002156 | 1.39E-05 | 379.4016 | 612 | 1 | 5.99E-03 |
| Inverse variance weighted | PSYCH_factor | ukb-d-30860_raw | 349 | -0.00355 | 0.000819 | 1.45E-05 | 280.5445 | 348 | 0.996716402 | 6.20E-03 |
| Inverse variance weighted | PSYCH_factor | met-d-VLDL_FC | 123 | 0.014031 | 0.003243 | 1.52E-05 | 116.4014 | 122 | 0.625964735 | 6.41E-03 |
| Inverse variance weighted | PSYCH_factor | ebi-a-GCST90095038 | 828 | 0.008313 | 0.001923 | 1.54E-05 | 509.7896 | 827 | 1 | 6.47E-03 |
| Inverse variance weighted | PSYCH_factor | ukb-d-30860_irnt | 359 | -0.01418 | 0.003323 | 1.97E-05 | 297.4664 | 358 | 0.991314042 | 8.11E-03 |
| Inverse variance weighted | PSYCH_factor | met-d-XL_VLDL_C_pct | 137 | 0.009926 | 0.002333 | 2.09E-05 | 132.8081 | 136 | 0.561413359 | 8.56E-03 |
| Inverse variance weighted | PSYCH_factor | ukb-d-30270_irnt | 731 | -0.00802 | 0.00192 | 2.95E-05 | 420.7296 | 730 | 1 | 1.17E-02 |
| Inverse variance weighted | PSYCH_factor | ukb-d-30130_irnt | 800 | -0.00746 | 0.001792 | 3.13E-05 | 459.9447 | 799 | 1 | 1.23E-02 |
| Inverse variance weighted | PSYCH_factor | ebi-a-GCST90092999 | 131 | 0.013001 | 0.003122 | 3.13E-05 | 106.0279 | 130 | 0.939108359 | 1.23E-02 |
| Inverse variance weighted | PSYCH_factor | ieu-b-4814 | 353 | 0.00122 | 0.000293 | 3.16E-05 | 206.1708 | 352 | 1 | 1.24E-02 |
| Inverse variance weighted | PSYCH_factor | ieu-a-300 | 144 | 0.0095 | 0.002284 | 3.19E-05 | 162.5443 | 143 | 0.125924711 | 1.25E-02 |
| Inverse variance weighted | PSYCH_factor | ebi-a-GCST90092817 | 64 | 0.016603 | 0.003996 | 3.26E-05 | 52.27062 | 63 | 0.830441842 | 1.27E-02 |
| Inverse variance weighted | PSYCH_factor | met-d-DHA_pct | 62 | 0.013494 | 0.003261 | 3.51E-05 | 57.63684 | 61 | 0.598545437 | 1.35E-02 |
| Inverse variance weighted | PSYCH_factor | ukb-b-20044 | 775 | 0.011395 | 0.002755 | 3.52E-05 | 416.3952 | 774 | 1 | 1.36E-02 |
| Inverse variance weighted | PSYCH_factor | ieu-a-89 | 718 | 0.007343 | 0.001779 | 3.66E-05 | 435.5689 | 717 | 1 | 1.40E-02 |
| Inverse variance weighted | PSYCH_factor | met-d-M_VLDL_L | 115 | 0.014508 | 0.003517 | 3.71E-05 | 130.0408 | 114 | 0.144594694 | 1.41E-02 |
| Inverse variance weighted | PSYCH_factor | ebi-a-GCST90018976 | 422 | -0.01368 | 0.003345 | 4.32E-05 | 329.3041 | 421 | 0.999663686 | 1.63E-02 |
| Inverse variance weighted | PSYCH_factor | ieu-b-38 | 947 | -0.00065 | 0.00016 | 4.37E-05 | 623.1718 | 946 | 1 | 1.64E-02 |
| Inverse variance weighted | PSYCH_factor | ukb-b-6704 | 769 | 0.011489 | 0.002829 | 4.87E-05 | 406.0781 | 768 | 1 | 1.82E-02 |
| Inverse variance weighted | PSYCH_factor | met-c-844 | 27 | 0.012008 | 0.002976 | 5.46E-05 | 20.55973 | 26 | 0.764317116 | 2.01E-02 |
| Inverse variance weighted | PSYCH_factor | ebi-a-GCST90092922 | 115 | 0.014208 | 0.003532 | 5.76E-05 | 133.2512 | 114 | 0.105086854 | 2.11E-02 |
| Inverse variance weighted | PSYCH_factor | ukb-b-19393 | 768 | 0.011554 | 0.002873 | 5.79E-05 | 429.5036 | 767 | 1 | 2.11E-02 |
| Inverse variance weighted | PSYCH_factor | ukb-d-30110_irnt | 913 | 0.006127 | 0.001552 | 7.93E-05 | 590.4594 | 912 | 1 | 2.84E-02 |
| Inverse variance weighted | PSYCH_factor | ukb-b-15590 | 761 | 0.011131 | 0.002825 | 8.17E-05 | 448.7771 | 760 | 1 | 2.91E-02 |
| Inverse variance weighted | PSYCH_factor | ieu-b-40 | 1066 | 0.010303 | 0.002656 | 1.05E-04 | 592.8029 | 1065 | 1 | 3.69E-02 |
| Inverse variance weighted | PSYCH_factor | ebi-a-GCST90025980 | 547 | 0.011105 | 0.002877 | 1.14E-04 | 417.2204 | 546 | 0.999988492 | 3.97E-02 |
| Inverse variance weighted | PSYCH_factor | met-d-VLDL_C | 119 | 0.011716 | 0.00306 | 1.29E-04 | 116.4009 | 118 | 0.524362596 | 4.48E-02 |
| Inverse variance weighted | PSYCH_factor | ebi-a-GCST90092866 | 135 | 0.009744 | 0.002545 | 1.29E-04 | 127.8448 | 134 | 0.633451606 | 4.48E-02 |
| Inverse variance weighted | PSYCH_factor | ebi-a-GCST90002391 | 375 | 0.01122 | 0.002935 | 1.32E-04 | 246.7939 | 374 | 0.999999939 | 4.55E-02 |
| Inverse variance weighted | PSYCH_factor | ebi-a-GCST90002401 | 1158 | 0.00569 | 0.001492 | 1.37E-04 | 711.2273 | 1157 | 1 | 4.69E-02 |
| Inverse variance weighted | PSYCH_factor | met-d-VLDL_L | 132 | 0.011782 | 0.003093 | 1.40E-04 | 99.53245 | 131 | 0.981416244 | 4.75E-02 |
| Inverse variance weighted | PSYCH_factor | ebi-a-GCST90002328 | 461 | 0.010967 | 0.002885 | 1.44E-04 | 282.0969 | 460 | 1 | 4.88E-02 |

**Supplementary Table 13.** Polygenic risk score based on aggregated data

| **Chr** | **PRS Score** |
| --- | --- |
| Chr1 | 6.79E-02 |
| Chr21 | 4.35E-02 |
| Chr5 | 2.97E-02 |
| Chr3 | 2.61E-02 |
| Chr18 | 2.31E-02 |
| Chr17 | 2.21E-02 |
| Chr13 | 1.79E-02 |
| Chr12 | 1.56E-02 |
| Chr16 | 1.45E-02 |
| Chr8 | 9.35E-03 |
| Chr22 | 6.90E-03 |
| Chr19 | -1.97E-01 |
| Chr2 | -1.26E-01 |
| Chr15 | -8.44E-02 |
| Chr7 | -3.26E-02 |
| Chr10 | -3.20E-02 |
| Chr20 | -2.66E-02 |
| Chr6 | -1.92E-02 |
| Chr14 | -1.62E-02 |
| Chr4 | -1.46E-02 |
| Chr9 | -1.37E-02 |
| Chr11 | -9.66E-03 |
